# Adding a Twist to Lateral Flow Immunoassays: A Direct Replacement of Antibodies with Helical Affibodies, from Selection to Application

**DOI:** 10.1021/jacs.4c17452

**Published:** 2025-03-26

**Authors:** Christy J. Sadler, Adam Creamer, Kim Anh Giang, Kevion K. Darmawan, André Shamsabadi, Daniel A. Richards, Johan Nilvebrant, Jonathan P. Wojciechowski, Patrick Charchar, Ross Burdis, Francesca Smith, Irene Yarovsky, Per-Åke Nygren, Molly M. Stevens

**Affiliations:** † Department of Materials, Department of Bioengineering, Institute of Biomedical Engineering Imperial College London, London SW7 2AZ, U.K.; ‡ Department of Physiology, Anatomy and Genetics, Department of Engineering Science, Kavli Institute for Nanoscience Discovery, University of Oxford, Oxford OX1 3QU, U.K.; § Department of Protein Science, AlbaNova University Center, KTH Royal Institute of Technology, Stockholm SE-114 21, Sweden; ∥ School of Engineering, RMIT University, Melbourne, VIC 3001, Australia; ⊥ Institute for Chemical and Bioengineering, ETH Zurich, 8093 Zürich, Switzerland

## Abstract

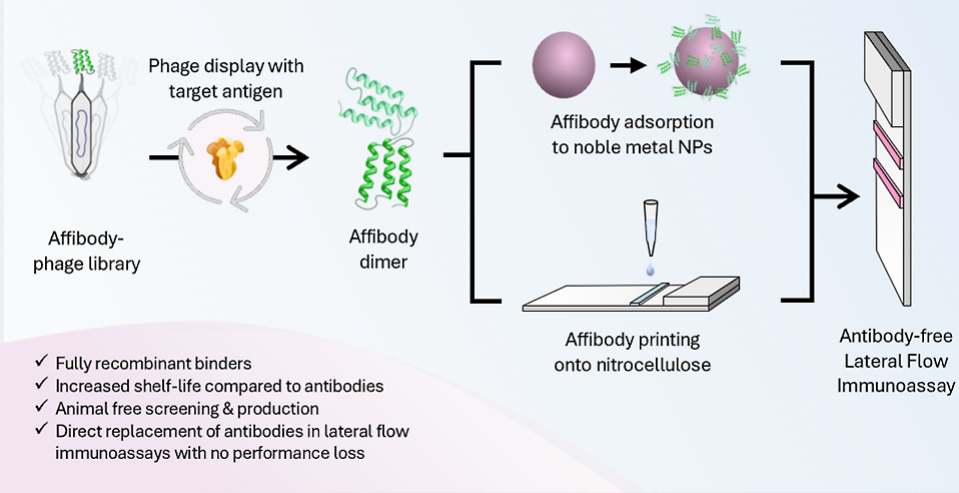

Immunoreagents, most commonly antibodies, are integral components of lateral flow immunoassays. However, the use of antibodies comes with limitations, particularly relating to their reproducible production, and poor thermal and chemical stability. Here, we employ phage display to develop affibodies, a class of nonimmunoglobulin affinity proteins based on a small three-helix bundle scaffold, against SARS-CoV-2 Spike protein. Subsequently, we demonstrate the utility and viability of affibodies to directly replace antibodies in lateral flow immunoassays. In addition, we highlight several physiochemical advantages of affibodies, including their ability to withstand exposure to high temperature and humidity while maintaining superior performance compared to their antibody counterparts. Furthermore, we investigate the adsorption mechanism of affibodies to the surface of gold nanoparticle probes via a His_6_-tag, introduced to also facilitate recombinant purification. Through molecular dynamics simulations, we elucidate the structural and physical characteristics of affibody dimers which result in high-performing detection probes when immobilized on nanoparticle surfaces. This work demonstrates that affibodies can be used as direct replacements to antibodies in immunoassays and should be further considered as alternatives owing to their improved physiochemical properties and modular design.

## 1 Introduction

Lateral flow immunoassays (LFIAs) have been one of the most impactful point-of-care diagnostic platforms, largely due to their low-cost, ease-of-use and scalability. LFIAs revolutionized the tracking and tracing of communicable diseases, particularly in low- and middle-income countries, and were a critical tool for disease detection and monitoring in the recent SARS-CoV-2 pandemic.^1^

Antibodies are one of the key components of most commercial LFIAs. In their native form, antibodies can be printed onto paper to form test (and control) lines, and physisorbed onto nanoparticles to produce detection probes, aiding a simple and scalable manufacturing procedure. However, LFIAs often rely on nonrecombinant monoclonal antibodies derived from hybridoma and/or polyclonal antibodies,^2^ which can present challenges relating to the reproducible production of reagents with well-defined characteristics.^3,4^ Furthermore, the common use of an animal host in the selection and production has raised important ethical concerns over animal welfare.^5,6^ These challenges have led in part to a recent report signed by 110 cosignatories calling for all commercial protein-binding reagents to be sequence-defined and produced recombinantly.^7^

To address some of the key concerns related to the use of nonrecombinant antibodies, a shift toward the use of nonanimal derived, sequence-defined affinity molecules has emerged.^6^ This includes the fully in vitro process for selection and production of antibodies, antibody derivatives [e.g., antigen binding fragments (Fab) and single-chain variable fragments (scFv)], aptamers, and scaffold-based affinity proteins (APs). The latter are small protein constructs, with a minimalist architecture, that can be developed to bind a desired target via combinatorial randomization of a set of surface-located positions, followed by in vitro selection using e.g. phage, bacterial or ribosomal display.^8^ Further, the emerging field of in silico binder design aims to eliminate the need for experimental selection processes entirely. However, the practical use in diagnostic applications is yet to be explored.

Affibodies are a subclass of APs and are of particular interest due to their small size (approximately 7 kDa and 58 amino acids, compared to approximately 150 kDa for antibodies), comparable binding affinities, and potential to regain activity after having been exposed to both extreme temperature (up to 95 °C) and nonphysiological pH (i.e., between pH 3 and 11).^9^ Furthermore, as the affibody structure lacks animal-derived Fc regions, concerns relating to cross-reactivity between human antibodies against animal-derived antibodies are reduced.^10^ Affibodies are selected via directed evolution (e.g., phage display) and produced via recombinant production (or using a peptide synthesizer), negating the need for any animal or animal model use in the selection or production processes.^9,11−13^ The minimalistic affibody architecture also allows for the straightforward production of multimeric constructs, such as the head-to-tail dimers demonstrated in this work, a difficult feat to achieve with antibody derivatives.^14^

Affibody research has primarily focused on in vivo theranostic applications.^15,16^ A relatively unexplored application of affibodies is in vitro diagnostics. They have been shown, in some settings, to be viable alternatives to antibodies in ELISAs,^17−19^ and protein microarrays.^20,21^ A recent study demonstrates that bovine serum albumin (BSA) conjugated affibody-like constructs function effectively as capture agents in LFIAs.^22^ However, to the best of our knowledge, the simultaneous use of affibodies as both capture and detection agents in LFIAs is yet to be explored.

As previously stated, full-length antibodies can be readily integrated into the LFIA design by membrane striping onto nitrocellulose and physisorbed onto nanoparticles (typically gold nanoparticles). The same cannot be said for antibody derivatives or the alternatives discussed above.^23^ For such affinity agents, the production of a functional test line is typically formed via protein biotinylation (with a streptavidin test line),^23,24^ fusion of an ‘anchor protein’,^22,23,25^ or direct chemical modification of the nitrocellulose membrane.^23^ The functionalization of nanoparticle detection probes with alternative affinity agents is equally nontrivial. The attachment of antibody-alternatives to nanoparticles can be achieved through covalent attachment (e.g., via amide coupling),^26^ or by utilizing noble metal–thiol interactions to mediate binding of affinity molecules (such as aptamers) to gold nanoparticles.^27−30^ The advantages of antibody-alternatives over full-length antibodies in LFIAs are therefore somewhat overshadowed by the manufacturing complexity when compared to standard LFIA manufacturing processes. This hinders the use of affinity molecules in low-cost, industrial-scale LFIA production.

**Figure 1:**
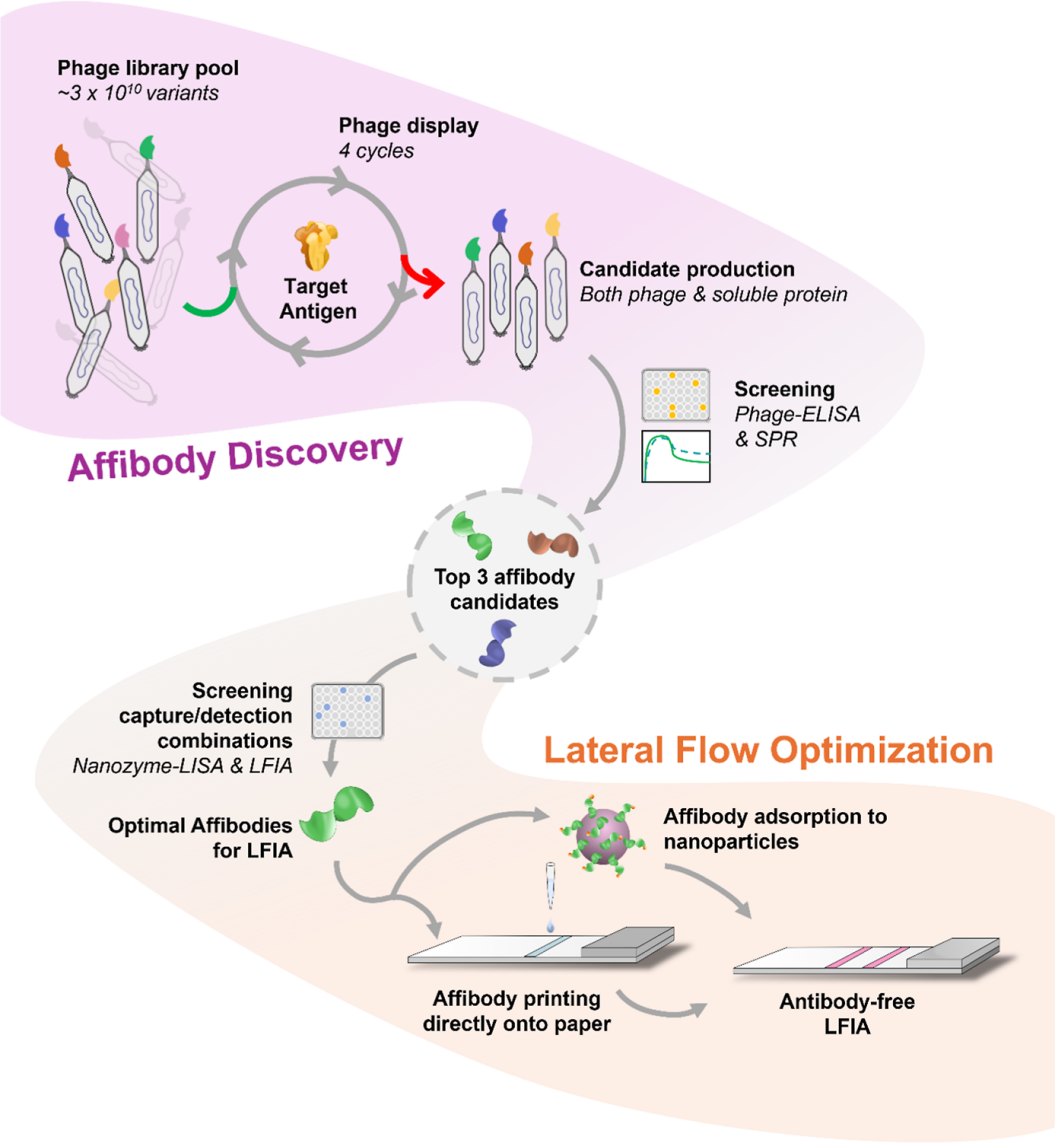
Schematic of the process of affibody discovery via phage display, followed by phage-ELISA and SPR screening. The top three affibody candidates were then produced recombinantly as head-to-tail dimers, followed by a further screening in nanozyme-LISA and LFIAs. The best performing affibodies were then taken forward to print directly onto nitrocellulose and adsorbed onto noble-metal nanoparticles to yield an antibody-free LFIA.

In this work, we showcase the development of an antibody-free LFIA from affibody discovery to implementation in a functional assay using SARS-CoV-2 Spike (S) protein as a model antigen (Figure 1). Specifically, we illustrate that affibodies can be used as a direct replacement to antibodies, without the need for chemical modification or alteration to industry-standard physisorption conjugation methods (with both catalytic platinum nanoparticles and gold nanoparticles) or nitrocellulose membrane preparations. Furthermore, we highlight the superior stability of affibody-based LFIA test strips in accelerated aging tests. We then constructed an antibody-free half dipstick LFIA to detect the presence of S protein in spiked human pooled saliva, demonstrating that affibody capture and detection affinity agents are compatible for use with a relevant sample matrix. We conclude this study by investigating the orientation of affibody dimer probes on noble metal nanoparticles, via molecular dynamics simulation, to help inform the future design of affinity proteins for LFIA applications.

## 2 Results and Discussion

### 2.1 Selection and Screening of Affibodies

**Figure 2:**
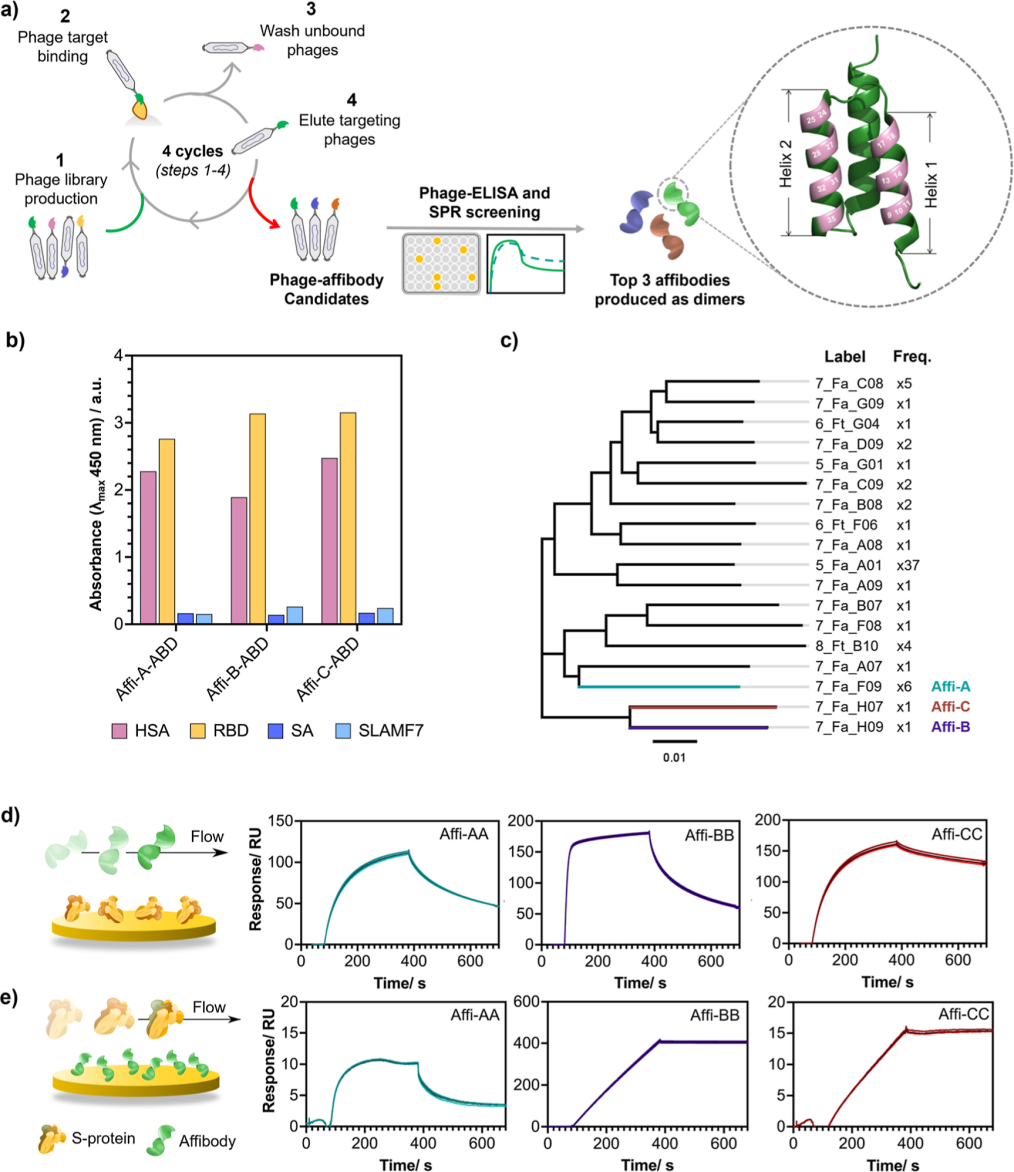
Affibody characterization and binding kinetics. (a) Schematic to illustrate the phage display process utilized to identify affibody candidates against the SARS-CoV-2 RBD or S1 protein. The affibody image was generated using Pymol (Schrodinger). (b) Monoclonal phage-ELISA absorbance values of Affi-A, B and C (displayed as fusion proteins with an albumin binding domain on the phage surface, which is used as a positive control for affibody display) when probed against human serum albumin (HSA), receptor-binding domain (RBD), streptavidin (SA) (negative control) and surface antigen CD319 (SLAMF7) (negative control), *n* = 1. (c) Phylogenetic tree illustrating the 18 unique affibody candidates identified from the phage display process. (d) SPR traces of each affibody when the S trimer was immobilized onto the SPR sensor surface and the dimeric affibody construct was injected over the sensor. (e) SPR traces of each affibody when the dimeric affibody was immobilized onto the SPR sensor surface and the S trimer protein was injected over the sensor, *n* = 3.

To identify suitable affibodies against the SARS-CoV-2 S protein, phage display was performed using a large (3 × 10^10^) affibody phage library. Recombinant receptor-binding domain (RBD) and the S1 subunit were selected as the protein targets. Briefly, each target was subjected to two parallel selection tracks, differing in the elution strategy (acid or trypsin digestion), and four selection rounds were performed with increased stringency for every round, including lower target concentrations and harsher washing conditions to remove non- or weak binders (Figure 2a). Following the final selection round, a monoclonal phage-ELISA was performed to identify target binding clones (Figures 2b and S1) that were subsequently subjected to DNA sequencing revealing a total of 18 unique candidate binders (Figure 2c).

Gel electrophoresis following soluble expression and affinity purification of affibodies as fusion proteins with a His_6_-tag and an albumin binding domain (ABD) showed the 18 affibody fusion constructs at the expected mass range (approximately 14–15 kDa) (Figure S2). Surface plasmon resonance (SPR) was utilized to assess the ability of the 18 constructs to bind to immobilized S1 or RBD protein. Three of the binders, denoted Affi-A, Affi-B and Affi-C, were selected for further evaluation due to their contrasting binding kinetics (Figure S3). The binder Affi-A showed the highest association response to immobilized RBD, and with a relatively slow dissociation. Affi-B showcased a distinct binding profile compared to the other affibodies, with both a slow association and a remarkably slow dissociation. Finally, Affi-C demonstrated a relatively fast association and slow dissociation, similarly to Affi-A, but with a lower overall measured response (Figure S3). The amino acid sequence for the three selected affibodies can be found in Table 1 (sequence information for all 18 affibodies can be found in Figure S4).

**Table 1:**
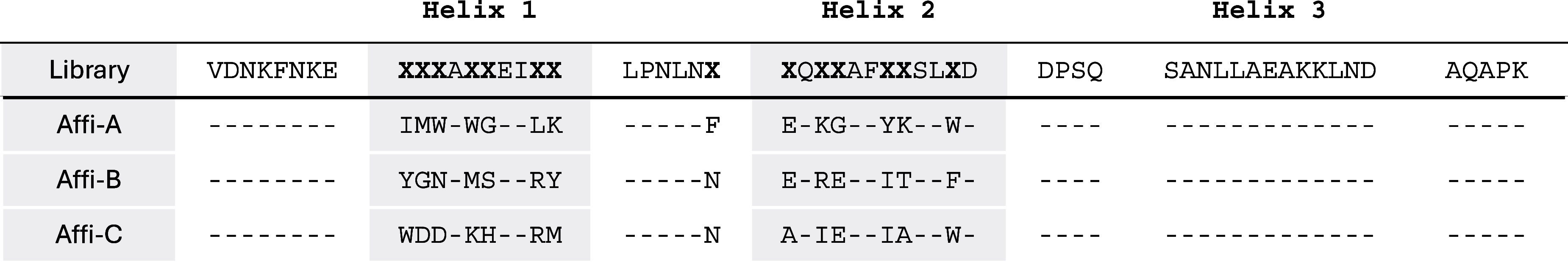
Amino Acid Sequence Alignment for the Affibodies Affi-A, Affi-B and Affi-C, Compared to the Library gene. Positions Marked with ‘X’ in the Library Gene Correspond to the Positions Subjected to Combinatorial Randomization in the Library

We anticipated that it should be possible to employ avidity effects to further increase the binding performance, due to the trimeric nature of the S protein diagnostic target. Thus, head-to-tail homodimerization was performed to link two affibodies in a tandem arrangement via a flexible (GGGSG)_3_ linker. In addition, affibody dimers containing a terminal cysteine (Cys) residue were included in this study to possibly further facilitate immobilization onto noble metal nanoparticle surfaces (vide infra). Two construct formats were therefore generated, His_6_-[affibody]-linker-[affibody]-Cys and [affibody]-linker-[affibody]-His_6_, thus generating the corresponding constructs denoted Affi-AA-Cys, Affi-BB-Cys and Affi-CC-Cys, as well as Affi-AA, Affi-BB and Affi-CC. Unless otherwise stated, all affibody constructs contain a terminal His_6_-tag.

We utilized SPR to compare the overall binding profiles of the different affibody in dimeric form. In LFIAs, the affibody affinity agents will be either affixed to nanoparticles or immobilized onto the nitrocellulose membranes. Therefore, the binding profiles to the S trimer antigen were assessed by SPR with the affibody both free in solution and affixed to a surface. In both cases, as the affibody and antigen were both multimeric, extracting kinetic values was not attempted. Initially, the S trimer antigen was immobilized on the SPR chip surface, followed by injection of the dimeric affibody (Figure 2d). By assessing the apparent kinetic profiles, Affi-CC demonstrated the slowest dissociation rate and a relatively fast association rate. Affi-BB was also shown to bind rapidly but exhibited a comparatively fast dissociation rate. In contrast, Affi-AA demonstrated the slowest binding association, with an intermediate level of dissociation. Affi-AA was also found to have the lowest response of the three affibodies tested, which was in direct contrast to the monomeric analogue (Affi-A, Figure S4). This could be explained by unfavorable internal interactions within the dimeric affibody at physiological pH (vide infra).

The reverse set up was then investigated, where the dimeric affibodies were immobilized on the SPR chip surface and S trimer antigen injected (Figure 2e). Injection of the S trimer antigen target over the three different affibody constructs resulted in two distinctive binding profiles. Affi-BB and Affi-CC demonstrated similar binding profiles, characterized by a very slow apparent dissociation rate, albeit with differing response levels. This may reflect an avidity effect via the formation of 3:3 complexes in which all three RBD units in the trimeric antigen became bound to closely immobilized affibody constructs. The observed dissociation rates are notably slower than those observed in the reversed binding format (Figure 2d), in which at most 2:2 complexes can be formed. However, Affi-AA showed a different binding profile, with a low binding response, and relatively high level of dissociation, compared to the other constructs. It is hypothesized that this is due to the sensitivity of this affibody toward the orientation of the epitopes within the injected trimeric S protein, whereby Affi-AA cannot form 3:3 complexes. As the three affibody dimers showed reasonable binding kinetics in both assay formats, they were all taken forward for consideration in LFIAs.

### 2.2 Screening of Immunoreagent Pairs in a Plate-Based Format

**Figure 3:**
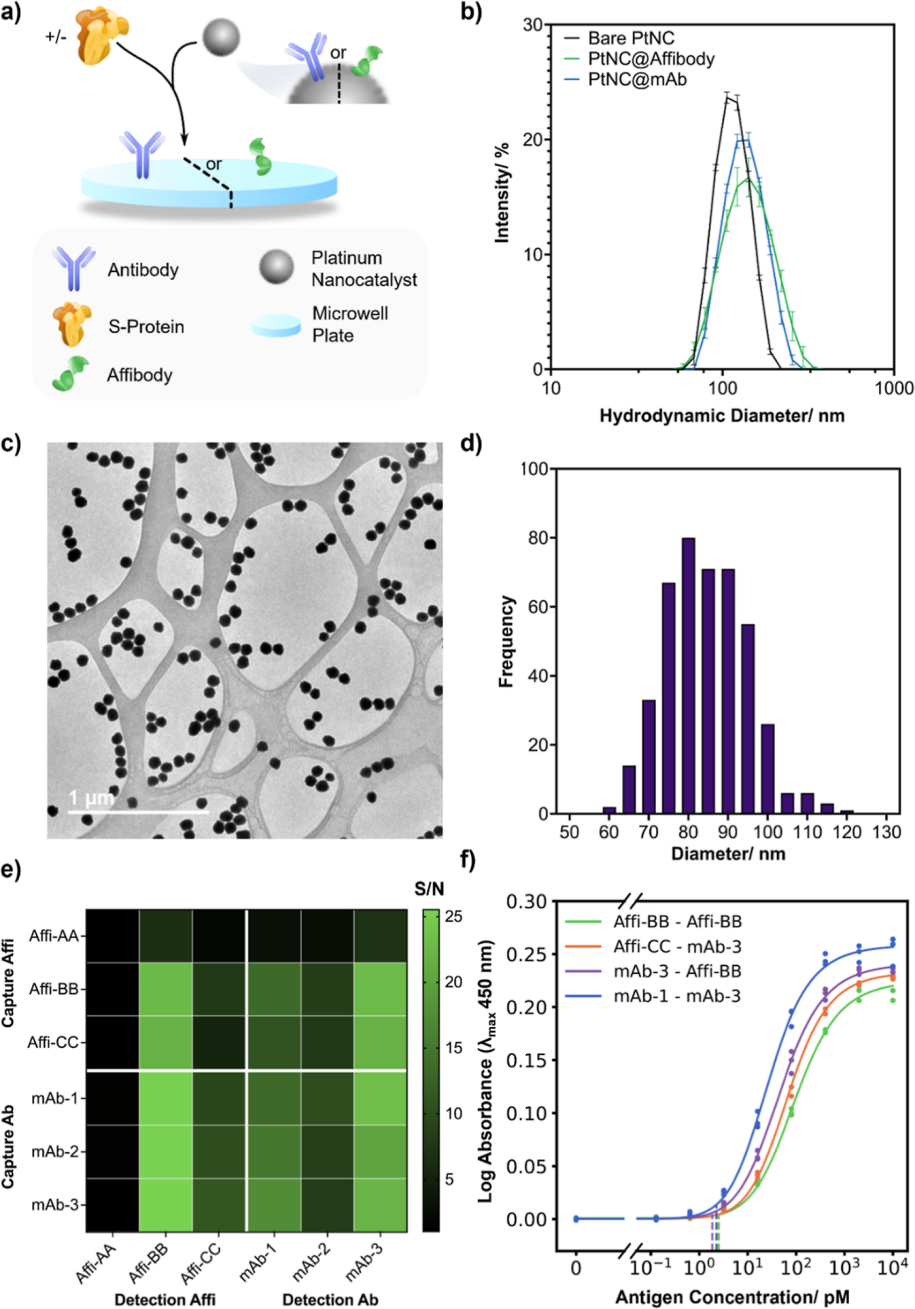
Screening of immunoreagent pair performance in plate-based assay format utilizing platinum nanocatalyst detection probes. (a) Illustration of the nanozyme-LISA format with affibody and antibody utilized as both capture and detection probes. (b) Hydrodynamic diameter of PtNCs pre- and postprotein functionalization as measured by DLS, mean ± S.D, *n* = 3. (c) TEM micrograph of synthesized PtNCs. (d) Histogram of PtNC diameter as measured from TEM micrographs, *n* = 435. (e) Heat-map of the signal-to-noise ratio (S/N) of screened affinity agent pairs with and without trimeric S protein (400 or 0 pM, signal and noise respectively). Data was obtained from the absorbance values at 450 nm, *n* = 2. (f) Sigmoidal curves of S protein dilution with the four combinations of antibody and affibody as capture and/or detection probes, where the dashed line represents the antigen concentration at the LOD. Presented here are the pair combinations with the lowest LOD. Data were obtained from the absorbance values at 450 nm, *n* = 3.

To achieve high performing LFIAs employing affibody affinity agents, it was first necessary to identify pairs of compatible capture and detection agents. As dimeric affibody constructs were unexplored for use as capture or detection probes in LFIAs, we initially combined these constructs with three commercially available monoclonal antibodies (labeled mAb-1, mAb-2 and mAb-3). Owing to the trimeric nature of the S protein antigen, we also explored the ability of the same affinity agent to be simultaneously used as both a capture and detection agent. This generated 81 unique affinity agent pairs (six homodimeric affibodies, including and omitting terminal cysteine residues, and three antibodies), which would be impractical to screen in a LFIA format. However, work is actively ongoing to develop automated, high-throughput screening platforms for the development of LFIAs.^31^ Therefore, a plate-based sandwich nanozyme-linked immunosorbent assay (nanozyme-LISA) was instead used due to its high-throughput nature (Figure 3a), while still preserving the nature of the detection probe, where the affinity agents are immobilized on the surface of nanoparticle probes. Further, by utilizing nanozymes in place of enzymes conjugated to secondary antibodies (such as antimouse horseradish peroxidase), we could avoid cross-reactivity when the capture and detection affinity agent originated from the same host species. Here, we utilized the peroxidase-mimicking platinum nanocatalysts (PtNCs), consisting of a gold nanoparticle seed (15 nm diameter) with a porous platinum shell, and with an average diameter of 84.6 ± 10.1 nm via TEM (Figure 3b–d, Table S1). The PtNC detection probes were decorated with mAbs and affibodies via physisorption, and capture affinity agents immobilized onto high-binding polystyrene surfaces via passive adsorption. The successful formation of a sandwich pair in a microwell could therefore be detected based on oxidation of 3,3′,5,5′-tetramethylbenzidine (TMB), resulting in an increase in absorbance at 450 nm.

Initial sandwich pair screening was performed by assessing the signal-to-noise ratio (S/N) generated in the presence and absence of S protein with each combination of mAbs and affibodies as both capture and detection agents (Figures 3e and S5). Affibodies were able to function comparably to mAbs as both capture and detection probes. Specifically, Affi-BB and Affi-CC both functioned well as capture agents, while Affi-BB also performed well as a detection agent. Conversely, Affi-AA performed poorly in all cases. It was found that affibodies equipped with a terminal cysteine residue performed comparably to the noncysteine counterparts when used as detection probes. However, for unknown reasons, the inclusion of the terminal cysteine residue hindered affibody performance as a capture probe when immobilized onto the high-binding polystyrene microwell plate (Figure S5).

Comparing the findings in Figure 3e to the SPR sensorgrams, the relative dissociation rates when the affibodies were immobilized on a surface (Figure 2e) appears to be the best predictor of performance in nanozyme-LISA with the slowest dissociation rate being more favorable. These findings suggest that, in nanoparticle-based sandwich assays, SPR could be a predictor of the performance when the affinity agent is immobilized. However, further work is needed to validate these preliminary findings.

From our initial nanozyme-LISA pair screening, the affinity agent pairs with the highest signal-to-noise ratios (S/N) were taken forward to assess their respective sensitivities. Affi-BB and Affi-CC were tested as capture probes, with both affibodies performing similarly when used alongside both affibody and mAb (Affi-BB and mAb-3, respectively) detection probes. The absorbance values were plotted as a function of S protein concentration, and a regression fitted to yield statistically driven limit of detection (LOD) values (Figures S6–S9). The sigmoidal curves for the top performing affinity agent pairs can be found in Figure 3f with their respective extracted figures of merit in Tables S2–S5. Although the absolute absorbance values were found to be higher for the antibody-only pairs, the computed LOD values between the affinity agent pairs were found to be remarkably similar and no statistical significance was found between the LOD values (Figure S10 and Table S6, using the antibody-only assay as a reference). This illustrates that affibodies could directly replace antibodies to produce functional detection and capture agents with comparable performance.

### 2.3 Screening of Immunoreagents in LFIAs

We hypothesized that the best performing pairs from nanozyme-LISA screening may not necessarily correlate to the top LFIA performers due to contrasting assay formats, such as prolonged incubation times and the nature of the solid-phase substrate (high binding polystyrene for nanozyme-LISA, nitrocellulose membranes for LFIAs). Therefore, a selection of the top performing pairs (25%) from the initial nanozyme-LISA screening (Figure 3e) were taken forward for further screening in a half-dipstick LFIA format. From the nanozyme-LISA screening assays, it was found the Affi-AA functioned poorly as both capture and detection probes. Owing to the lack of appreciable binding in these assays, Affi-AA was excluded from the LFIA screening study. However, Affi-BB and Affi-CC were taken forward as both capture and detection probes.

**Figure 4:**
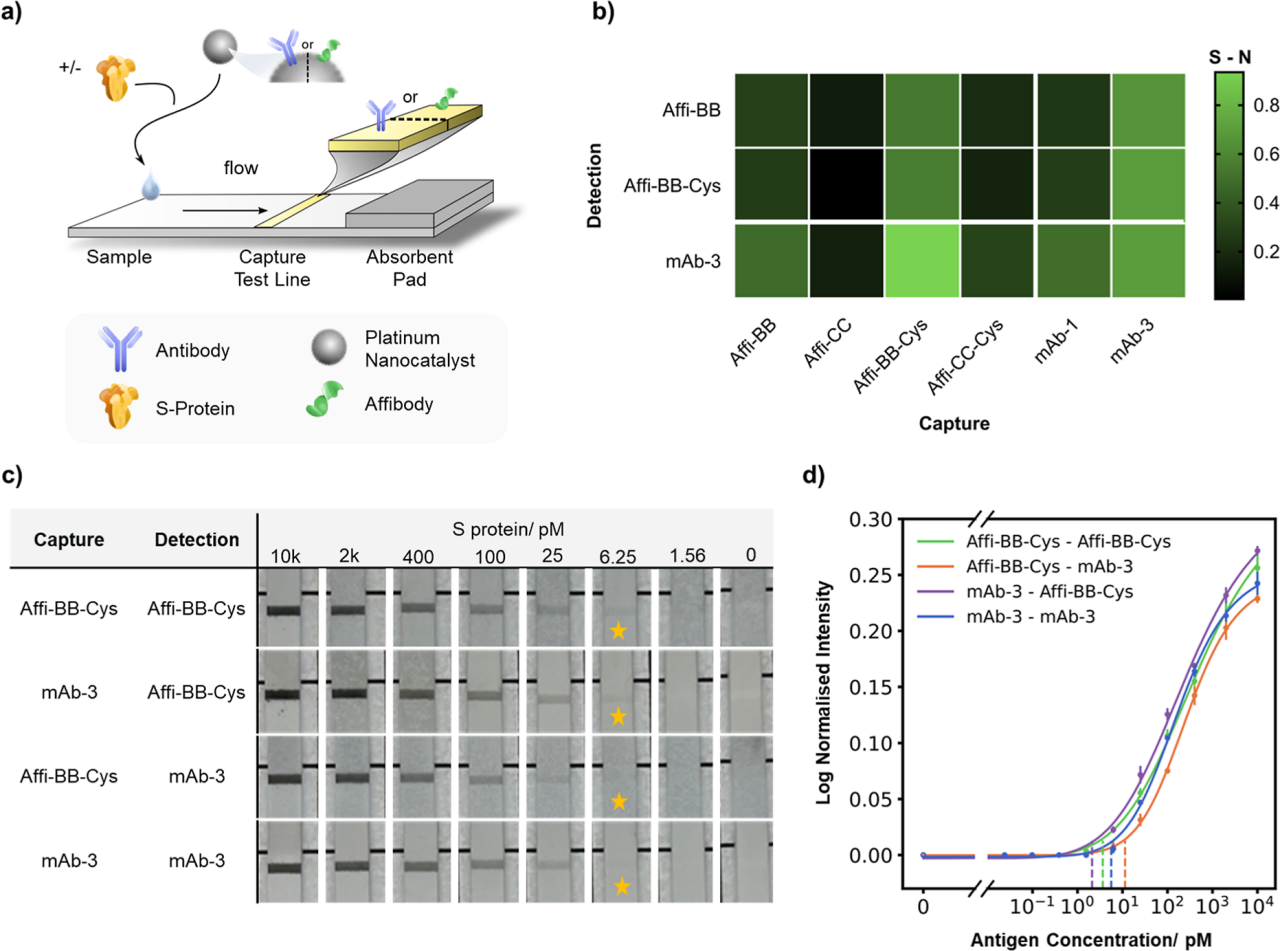
Screening of immunoreagent pair performance in paper-based assay format utilizing platinum nanocatalyst detection probes. (a) Illustration of the format of a PtNC-based LFIA with affibody and antibody as both capture and detection probes. (b) Heat-map of the signal-minus-noise (S–N) of screened affinity agent pairs with and without trimeric S protein (400 or 0 pM, signal and noise respectively). Data was obtained from the test line intensity extracted from the pixel intensity of the image, *n* = 2. (c) Representative photographs of PtNC-based LFIA using a serial dilution of the S protein antigen for the four combinations of antibody and affibody as capture and/or detection probes. Yellow star denotes the visual LOD (vLOD), defined as the lowest concentration whereby a test line is visible and can be distinguished from the background. (d) Sigmoidal curves of S protein dilution with the four combinations of antibody and affibody as capture and/or detection probes, where the dashed line represents the antigen concentration at the LOD, *n* = 3.

LFIA sandwich pair screening was performed utilizing PtNCs to assess the affinity agent pairs with maximal signal and minimal noise. The LFIA screening was performed with a catalytic signal amplification step (using CN/DAB as the substrate), as reported previously (Figure 4a).^24^ The signal and noise values for each pair were plotted as signal-minus-noise (denoted S–N, Figure 4b). After catalytic amplification, a negative intensity was observed at the test line, termed a ghost line. This is thought to originate from differences in hydrophobicity between the nitrocellulose membrane and test line. In these cases, the test line intensity was normalized to zero (no nonspecific binding). In all combinations of affibody and mAb affinity agents, and as both capture and detection, the Affi-BB-Cys and mAb-3 combination was found to be optimal. No significant difference in performance was observed between affibody constructs with and without a terminal cysteine when used as detection probes alongside PtNCs, in agreement with our plate-based screening. However, in direct contrast to the performance in nanozyme-LISA, the inclusion of the terminal cysteine residue in the homodimeric affibody constructs resulted in a significant increase in signal when used as capture (via nitrocellulose striping). The influence of cysteines on affibodies as capture probes requires further investigation. The functionalization of PtNCs with affibodies or mAbs was then optimized to ensure a fair comparison and eliminate any nonspecific binding on the test line (i.e., signal with no antigen) and/or nitrocellulose membrane staining (i.e., background signal). The following parameters were screened: conjugation pH, affinity agent-nanoparticle ratio and blocking reagents (Figures S11 and S12). The conditions chosen for all conjugations were pH 7 with a blocking solution consisting of 1 wt % poly(vinylpyrrolidone) and 1 wt % β-casein. Our previous work illustrated that β-casein was a viable blocking agent for use alongside PtNCs, hence it was utilized over other common blocking proteins, such as BSA.^24^

After optimization of the affibody and mAb detection probes, the sensitivity of the respective sandwich pairs to detect the S protein antigen in DPBST (Dulbecco’s PBS with 0.05 v/v % Tween 20) was assessed. Figure 4c shows representative images of the LFIA strips for each pairing (see images of the further repeat in Figure S13). It was found that the visual limit of detection (vLOD) was comparable for all four affinity agent pairs, at ca. 6 pM. The test line intensity plotted as a function of S protein concentration can be found in Figure 4d with extracted statistical parameters, in Table S7. The computed LOD for mAb or affibody only affinity agent pairs demonstrated no statistical significance (5.60 and 3.33 pM, respectively). This highlights that affibodies are viable alternatives to antibodies as both capture and detection probes in LFIA.

### 2.4 Antibody-free LFIA in Spiked Human Saliva

Although PtNCs offer additional assay sensitivity due to the catalytic enhancement steps, gold nanoparticles (AuNPs) are by far the most common detection probe in LFIAs.^1^ Therefore, we investigated the use of affibody constructs with commonly used 40 nm AuNPs in pooled human saliva. Here we chose to utilize the best capture and detection affibody pair from the PtNC study, with the assumption that this trend in performance would be preserved when moving to AuNPs, to showcase the versatility of affibodies across nanoparticle types.

**Figure 5:**
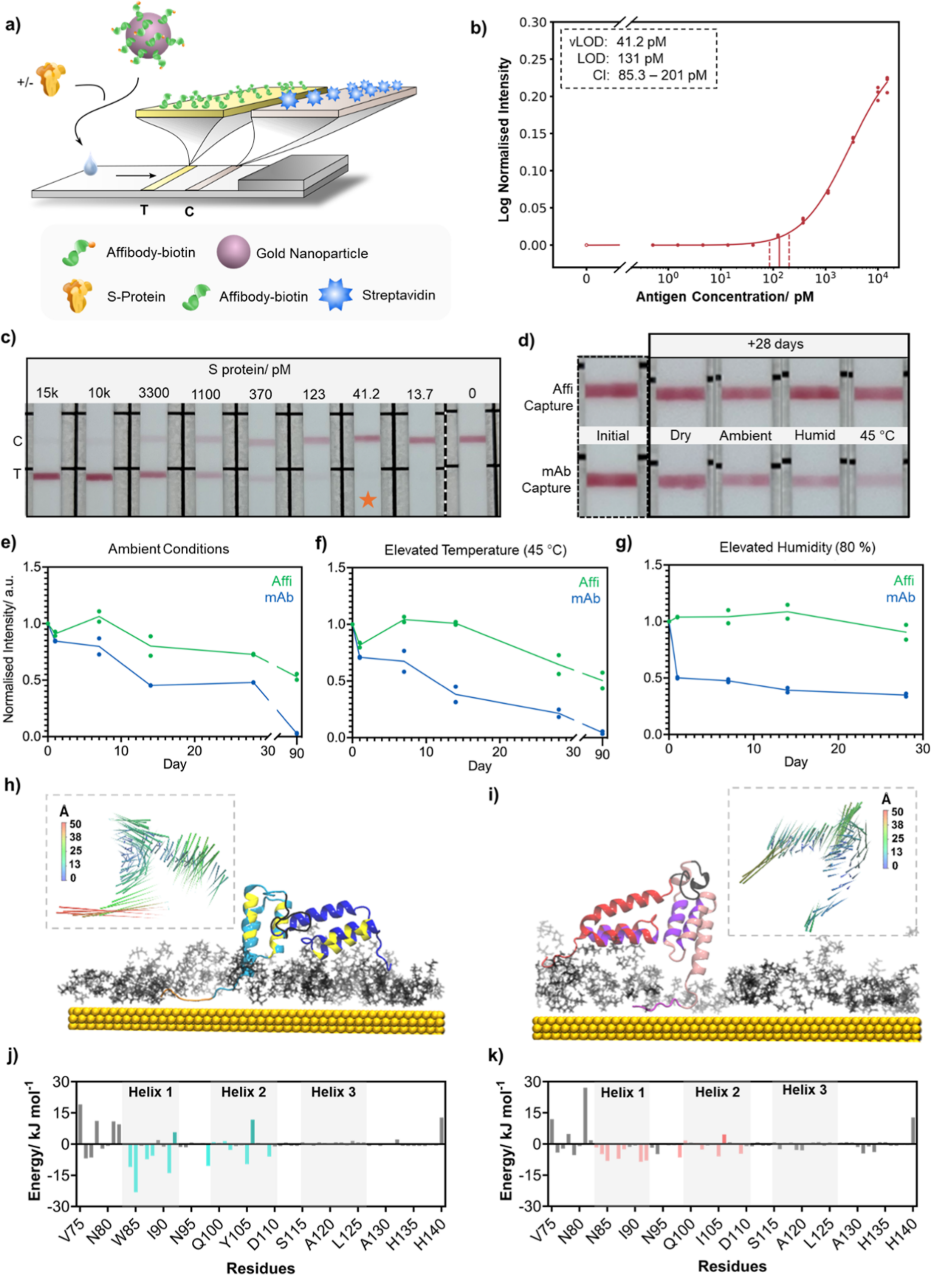
Proof-of-concept antibody-free LFIA using gold nanoparticles. (a) Illustration of the components of the antibody-free LFIA. (b) Sigmoidal regression curves of S protein dilution with overlaid visual and statistical limit of detection (vLOD and LOD, respectively) with confidence interval (CI), *n* = 3. (c) Representative image of the strips used to plot the sigmoidal curve. (d) Representative images of strips with Affi-BB-Cys or mAb-3 capture test lines, before and after 28 days stored in a desiccator (dry), at ambient conditions, elevated humidity (humid) or elevated temperature (45 °C). (e–g) Test line intensities as a function of storage time under each condition. Intensity values were obtained from the test line pixel density normalized to the grid line in each image. The extracted values were then normalized to the intensity value at day 0, *n* = 2. (h,i) Exemplar MD simulated configurations of Affi-AA (h) and Affi-BB (i) on the surface of citrate coated Au(111), with N-terminal affibody shown in blue/red colors and the C-terminal affibody shown in cyan/pink colors, respectively. Mutated affinity modulating residues between the two constructs shown in yellow for Affi-AA and purple for Affi-BB. Gold surface atoms shown in yellow; citrate molecules in gray. Water and ions are not shown for clarity. The inset porcupine plots illustrate the magnitude of the protein residue dynamics, representing 67% of the movement for Affi-AA and 69% of the movement for Affi-BB, with respective cosine values of 0.37 and 0.46. (j,k) Average residue contributions in kJ mol^–1^ to binding of the RBD of SARS-CoV-2 S protein to A-His_6_ (j) and B-His_6_ (k). The mutated affinity modulating residues between dimeric affibody constructs are colored in cyan for A-His_6_ and pink for B-His_6_, with unfavorable interactions highlighted by the darker respective color shades. Favorably interacting residues in A-His_6_: M84, W85, W87, G88, L91, F98, Y105, and W109; in B-His_6_: G84, N85, M87, R91, Y92, N98, I105, and F109. Unfavorably interacting residues in A-His_6_: K92 and K106; in B-His_6_: T106.

Affi-BB-Cys, the best performing affibody in LFIA, was used as a capture reagent for the design of an antibody-free half-dipstick LFIA (Figure 5a). In this case, the detection affibodies were produced with a C-terminal biotin (added via an AviTag), allowing for the inclusion of a control line consisting of polystreptavidin. The biotinylated affibody homodimer (Affi-BB-Biotin) was incubated with 40 nm AuNPs, following a modified method often employed with antibodies.^32^ As before, conjugation conditions were optimized to maximize signal and eliminate noise (Figure S14). The optimal conjugation conditions were found to be pH 7.6 and 2 w/v % β-casein as a blocking agent. Bare AuNPs and functionalized AuNPs were characterized to determine hydrodynamic diameter, concentration, and surface charge (Figure S15 and Table S8) and showed an increase in hydrodynamic diameter and surface charge on functionalization.

Human pooled saliva (spiked with S protein) was diluted into an optimized ratio of running buffer (ratio of 3:2, respectively) to reduce viscosity and maximize the volume of sample that could be used. The performance of the antibody-free half-dipstick LFIA was assessed using a serial dilution of S protein, where the quoted concentration of S protein represents the final concentration of antigen in the sample. Test line intensities were plotted as a function of S protein concentration (Figure 5b). To determine the statistical LOD, test line intensities were fitted with a regression model. Representative images of LFIA strips can be seen in Figure 5c and additional images in Figure S16. The production of an affibody-based LFIA validates that affibodies are compatible for use alongside the industry-standard AuNPs and can successfully detect the presence of a target antigen in a complex human sample.

### 2.5 LFIA Aging Study

An aging study was performed to assess the stability of affibody and mAb capture probes in a half-dipstick LFIA format when stored under a range of aberrant conditions compared to the standard LFIA storage conditions (stored in a dry environment, ca. 23% humidity and at room temperature, 20 °C). The conditions included: ambient conditions (room temperature, 21 °C and humidity, 56%), an elevated humidity chamber (room temperature, 21 °C and humidity, ca. 80%), and elevated temperature (45 °C oven, at ambient humidity, ca. 22%).

The performance of affibody and mAb LFIA strips stored under the different conditions were assessed via comparison of the test line intensity generated in the presence of S protein when utilizing a LFIA strip stored under optimal conditions (dry, in a desiccator). It should be noted that no noise (signal without antigen) was observed throughout the testing. The performance of each capture probe was assessed as a function of time in each condition (with time points at day 1, 7, 14, 28 and 90). These were compared to the initial performance directly after fabrication (day 0, Figure S17). Figure 5d shows representative images of strips stored for 28 days under the conditions described above, with all images shown in Figure S18. As expected, under standard storage conditions, there was no significant decrease in performance for affibody or mAb capture probes. Whereas, under the challenging conditions, a relative drop in performance was observed for mAb. Quantitative analysis of test line intensities as a function of time (Figure 5e–g) revealed an immediate loss of activity for mAb when stored under ambient conditions, high humidity and at elevated temperature. In contrast, affibody test lines showed a superior ability to function in all storage conditions. In particular, affibodies show a remarkable resistance to humid environments with a negligeable drop in performance over the full 28 days (Figure 5g). Affibodies are known to be resilient to long-term storage,^9^ and it is thought that the high humidity levels could allow for refolding of the affibody on the nitrocellulose membrane. An additional 90 day time point was recorded for ambient conditions and elevated temperature and showed a complete loss of performance for mAb whereas affibodies retained function. Aging for 90 days at 45 °C is approximately equivalent to 12 months at room temperature (estimated using the Arrhenius reaction rate function, which estimates that an increase of 10 °C will double the reaction rate).^24,33^

The superior performance of affibody capture probes under challenging storage conditions, when used as direct antibody replacements, clearly demonstrates the advantage of affibody integration into LFIAs. In particular, this aging study demonstrates the advantages of integrating affibodies into point-of-care devices intended for use in low-resource settings, whereby specialist storage conditions add unwanted cost and are less practical to implement.

### 2.6 Influence of Hexahistidine-Tag on Affibody Dimer Performance

As discussed above, prior efforts to immobilize antibody mimics onto nanoparticle surfaces typically involve a chemical modification step. However, this was not the case for affibody dimers, which could be simply physisorbed onto nanoparticle surfaces, yielding high-performing detection probes. Through the screening of affibody homodimer constructs in nanozyme-LISAs and LFIAs, we noted that the inclusion or omission of terminal cysteine had little to no effect on the performance when anchored on the nanoparticle surface. This observation indicated that the terminal cysteine was not the only functional amino acid residue responsible for the binding of affibody probes to nanoparticle surfaces. Histidine has also been reported to be a chelator to noble metal nanoparticles; therefore we hypothesized that the coordination of the affibody was also primarily dictated by the hexahistadine (His_6_) tag.^34−38^

In order to investigate histidine coordination to the nanoparticle surfaces, we produced Affi-BB with the absence of a His_6_-tag (Figure S19). The resulting affibody was conjugated to PtNCs and AuNPs and the performance on LFIA was compared to the His_6_-tagged Affi-AA and Affi-BB. Affi-BB without tag performed poorly when following the same physisorption protocol used for the His_6_-tagged Affi-BB (Figure S20). This suggested that either the affibody could not bind to the nanoparticle surface or was immobilized on the surface in an unfavorable manner for target binding. However, both affibody-functionalized nanoparticles (with and without the His_6_-tag) showed a similar change in surface charge (i.e., a more positive zeta potential) when compared to unfunctionalized nanoparticles (Table S9). This suggests that the omission of the His_6_-tag still resulted in affibody-coated nanoparticles but with an unfavorable affibody orientation. As expected, from the nanozyme-LISA pair screening (Figure 3b), Affi-AA performed poorly as a detection probe with both nanoparticle types (Figure S20).

Dimeric affibodies were chosen to use in study due to the improved binding profiles resulting from avidity effects with the multimeric antigen. We wanted to investigate whether dimeric constructs provided any performance enhancement as detection probes in LFIA format. Therefore, the ability of monomeric, His_6_-tagged affibody constructs to function in LFIAs when adsorbed onto AuNP and PtNC detection probes was also investigated (Figure S20). Monomeric Affi-A and Affi-B conjugated AuNPs produced detection probes that both performed relatively poorly, compared to their dimeric analogues. Therefore, constructs being dimeric and His_6_-tagged was critical to their excellent performance as detection probes which prompted further investigation using molecular dynamic simulations.

### 2.7 All-Atom Molecular Dynamics (MD) Simulations

In light of the aforementioned experimental findings, it is evident that various aspects of the affibody construct design, beyond the mutated affinity modulating residues within the affibody, are important for successful implementation as affinity agents in LFIAs. The experimental findings warranted further *in silico* investigations to shed light on the molecular mechanisms of the novel affibody dimers for LFIA applications. All-atom MD simulations of the best (Affi-BB) and worst (Affi-AA) performing detection constructs were employed to explain possible causes of the observed performance discrepancies. Specifically, the modeling aimed to compare the structural and dynamic properties of [affibody]-linker-[affibody]-His_6_ constructs (ordered from N to C terminus, as produced) in aqueous solution and immobilized on gold, to highlight the differences in the properties of the affibody dimers as observed in SPR and LFIAs. The AuNPs used in this study have a truncated octahedral morphology, with predominantly Au(111) facets.^39,40^ Therefore, citrate-capped flat Au(111) surfaces were used to model the nanoparticle surface.

Analysis of preferred conformations of dimeric affibody constructs in aqueous solution revealed that in all N-terminal affibodies (i.e., no His_6_-tag) the mutated affinity modulating residues prefer to face outward and are available for an antigen to bind, while in the C-terminal affibodies (i.e., with His_6_ tag) the mutated affinity residues appear to engage in the interactions with the linker (see Figure S21, refer also to Table S10 for further discussion). In contrast, when the dimeric affibodies were immobilized on the surface of citrate-coated Au(111), the mutated affinity modulating residues situated at the N-terminus were mostly found to orientate down toward the Au(111), limiting their capacity to interact with the target (RBD). However, the helical bundle of the C-terminal affibody was consistently found to be orientated perpendicular to the Au(111) surface, providing steric access for potential RBD binding. The advantages of such orientation are corroborated by the principal component analysis (PCA) in the form of porcupine plots of the protein backbone alpha-carbon atoms (Cα) that depict the movement of the residues along the first eigenvector (PC1) (Figures 5h,i; refer to Figure S22 a–c for the top 10 eigenvector values, PC2 & PC3, individually). This analysis indicates that while the individual mobility of residues in the C-terminal affibody indicates that Affi-AA is slightly more dynamic than Affi-BB, both affibodies are stable on the surface. The observed affibodies surface orientations and the resultant exposure of the mutated affinity modulating residues plausibly explains the differences between the properties of the dimeric affibody molecules in nanozyme-LISA or SPR assays and those of LFIA. Namely, in solution, the enhanced accessibility of the mutated affinity modulating residues within the N-terminal affibody facilitates the antigen interactions with the dimeric affibodies (Figure S23). Additional information detailing quantitative metrics that describe the steric availability of the mutated affinity modulating residues, including distance (Table S11) and contact analyses (Table S12), as well as exemplary representations of other MD sampled orientations of the affibodies on the surface of Au(111) (Figure S24) are elaborated in the Supporting Information.

MD simulations were also performed to investigate the C-terminal affibody complex with the RBD of the SARS-CoV-2 S protein in solution. The His_6_-tagged affibody was selected for this task over the His_6_-tag free counterpart because it was found to be more available for the antigen binding by the protein–surface simulations described above. Molecular mechanics Poisson–Boltzmann surface area (MM-PBSA) calculations illustrated that both affibodies (A and B) favorably bind to the RBD, with Affi-B binding ca. 30% more strongly as indicated by the higher binding energy (see approximate calculated Δ*G* in Table S13, Figure S25). Upon decomposing the binding energy into contributions from favorable residues, it was found that both A-His_6_ and B-His_6_ have eight mutated affinity modulating residues exhibiting favorable interactions to the RBD (Figure 5j, k). Further, two mutated affinity modulating residues, lysine K92 and K106, within Affi-A could hinder the formation of the protein–protein complex (Figure 5j) while only one residue, T106, was identified to potentially impede the binding of the B-His_6_ to the RBD of SARS-CoV-2 (Figure 5k). These findings are consistent with previous studies which showed that negatively charged proteins interact more favorably with the RBD of SARS-CoV-2 S protein than those charged positively.^41,42^ Additionally, hydrogen bonding analysis showed that B-His_6_ forms a higher number of hydrogen bonds to the RBD compared to A-His_6_ (Figure S26). Further work is required to validate these findings experimentally, including epitope mapping and resolving crystal structure of affibody-RBD complexes.

The MD simulations highlight the molecular mechanisms behind the contrasting behavior of Affi-AA and Affi-BB when utilized as detection probes in LFIAs. The in-solution and surface immobilized structures of Affi-AA show contrasting conformations, which may explain the observed difference in response for the SPR assays whereby the affibodies were in the mobile or stationary phase (Figure 2d,e). The simulations suggest that the N-terminal affibody monomer has a lesser influence on antigen binding ability since it has a propensity to orientate toward the gold surface, while the C-terminal affibody unit is more sterically accessible; thereby, facilitating more favorable interactions with the antigen. Further, the electrostatic and hydrogen bond analyses of the binding free energy contributions to antigen binding shows more favorable interactions for the C-terminal affibody monomer of Affi-BB than those of Affi-AA. Coupling these structural considerations with the insight into kinetic profiles (from SPR screening) could contribute to future design of even higher affinity proteins.

## 3 Conclusions

This work demonstrates the successful discovery, development and implementation of dimeric affibody constructs as both capture and detection agents in paper-based immunoassays. We illustrate how affibodies can be generated and selected using phage display, with phage ELISA and SPR as screening platforms. We then evaluated three candidate binders using a high-throughput, plate-based assay to determine suitable capture and detection probe combinations for LFIAs. We further demonstrate that affibodies could be utilized as direct replacements to antibodies when used alongside commercially viable nanoparticle conjugation and nitrocellulose membrane preparation methods, with no reduction in performance. We showcase this through the development of an antibody-free LFIA utilizing AuNPs in pooled human saliva. This highlighted the compatibility of affibodies to function in human saliva. However, further work is required to investigate the broad applicability of affibodies to function in other human matrices (such as whole blood, serum, and plasma). This includes the assessment of libraries of affibodies against a variety of antigen targets. The superior stability of affibody capture probes under elevated temperature and high humidity, compared to antibodies, is also highlighted. Additionally, we reinforce the existing literature understanding on the ability of His_6_-tagged proteins to bind to noble metal nanoparticle surfaces and promote favorable affibody orientation. Finally, MD was employed to provide an insight into the critical structural features and physical properties which result in high-performing probes on nanoparticle surface. This work demonstrates that affibodies are a viable alternative to antibodies in lateral flow immunoassays, owing to their matched performance, improved physiochemical properties and modular design.

## 4 Experimental Section

### 4.1 Identification of Affibodies against SARS-CoV-2 Spike Antigens

The identification of affibodies binding to SARS-CoV-2 S protein was performed as described in Giang et al.^43^ Briefly, a naive phage stock was prepared from an M13 phage library based on the phagemid vector pAffi-1.^44^ Four cycles of biopanning were performed using recombinant, biotinylated S1, provided by Helena Persson Lotsholm, DDD (SciLifeLab), (Biotinylated SARS-CoV-2 Spike RBD Protein, His, AviTag, cat. no. S1N-C82E8), or RBD protein (Biotinylated SARS-CoV-2 Spike RBD Protein, His, AviTag, cat. no. SPD-C82E9) as the targets, with increasing stringency for each cycle to enrich for target binders.

Monoclonal phage stocks generated from the final selection cycle output were diluted 1:3 with PBS-T (150 mM NaCl, 8 mM Na_2_HPO_4_, 2 mM NaH_2_PO_4_·H_2_O, pH 7.4, 0.05 v/v % Tween20 [Sigma]) and incubated with 10 μg mL^–1^ biotinylated S1 or RBD protein, 20 μg mL^–1^ human serum albumin (HSA; [Sigma]) (for assessment of proper display of the expression cassette containing the tripartite fusion protein consisting of an affibody, an albumin binding domain and the truncated protein 3), 10 μg mL^–1^ streptavidin (for assessment of binding to streptavidin, present on the streptavidin-coated paramagnetic beads used during the selection) or 10 μg mL^–1^ unrelated control protein (human SLAMF7, Acro Biosystems) coated on MaxiSorp ELISA plates (Clear Flat-Bottom Immuno Nonsterile 384-Well Plates, ThermoFisher Scientific) for 1 h at room temperature (RT) with slow shaking. Following three washes with PBS-T, HRP-conjugated α-M13 antibody (Sigma) diluted 1:5000 in PBS-T was added to the plate and incubated for 30 min at RT with slow shaking. After additional two washes with PBS-T and one wash with PBS, α-M13-HRP was detected by the addition of TMB substrate (TMB Substrate Kit, ThermoFisher Scientific). The reactions were stopped by 1:1 addition of 2 M H_2_SO_4_ after 15–25 min. Absorbances were measured at 450 nm using a CLARIOstar microplate reader (BMG Labtech). Candidates with a high HSA signal and relatively high signal to S1 or RBD compared to the streptavidin and unrelated target controls were considered as ELISA-positive, which were subsequently sequenced by Sanger sequencing (PlateSeq, Eurofins Genomics). Unique clones were identified using the Translation Alignment function with default settings and a phylogenetic tree was constructed for the same sequences, using the Geneious Tree Builder function with default settings (Geneious Prime, https://www.geneious.com). The phagemids containing the candidate gene variants were recovered using QIAprep Spin MiniPrep Kit (QIAGEN) and following the manufacturer’s protocol.

### 4.2 Analyses of S Protein Binding Candidates as His_6_-Affibody-ABD Fusion Proteins

DNA fragments encoding 18 ELISA-positive and unique S protein binding candidates were subcloned using In-Fusion HD Cloning Kit (Takara Bio) as monomeric affibody constructs with an N-terminal His_6_-tag and a C-terminal albumin binding domain (ABD).^45^ Cloned constructs were verified using Sanger sequencing (Eurofins Genomics) and expressed as soluble gene products in *Escherichia coli* BL21 (DE3) (Novagen). Harvested cells were subjected to lysis for 2 h at 150 rpm and 37 °C in lysis buffer (7 M guanidinium hydrochloride, 47 mM Na_2_HPO_4_, 2.65 mM NaH_2_PO_4_, 10 mM TRIS-HCl, 100 mM NaCl, pH 8). Lysate supernatants were purified under denaturing conditions using 3 mL HisPur Cobalt IMAC resin (ThermoFisher Scientific) per construct using batch method purification (sample and resin are incubated together in tubes with end-overend rotation and lysate supernatant sample, wash and elution fractions are separated from the resin through centrifugation). After wash (wash buffer: 7 M guanidium chloride, 46.6 mM Na_2_HPO_4_, 3.4 mM NaH_2_PO_4_, 300 mM NaCl, 10 mM imidazole, pH 8) and elution (elution buffer: 6 M urea, 50 mM NaH_2_PO_4_, 100 mM NaCl, 30 mM glacial acetic acid, 70 mM sodium acetate, pH 5), samples were buffer exchanged to PBS using PD-10 desalting columns (Cytiva). Absorbance was measured at 280 nm to calculate concentrations. Purity was assessed by SDS-PAGE.

Purified, soluble His_6_-affibody-ABD fusion proteins were screened for target binding by SPR using a Biacore 8K instrument (Cytiva). S1 and RBD proteins were immobilized as ligands on a Series S Sensor Chip CM5 (Cytiva) by separately attaching them to the carboxymethylated dextran surface on the second flow cell of two channels by amine coupling, using the manufacturer’s instructions, with final immobilized ligand levels 1464 and 968 response units (RU), respectively. The first flow cell of each channel was activated and deactivated for use as reference surfaces. The 18 His_6_-affibody-ABD fusion proteins were diluted in running buffer PBS-T to 200 nM and injected over the channels at 4 °C and at a flow rate of 30 μL min^–1^. Channels were regenerated with 10 mM HCl after each sample.

### 4.3 Analyses of S Protein Binding Candidates as Homodimeric Affibody Constructs

In-Fusion HD Cloning Kit (Takara Bio) was used to subclone DNA fragments encoding Affi-A, Affi-B and Affi-C as homodimeric constructs with a C-terminal His_6_-tag. All constructs were cloned with a sequence encoding for a flexible (GGGSG)_3_ linker between the affibody sequences. The constructs were then sequence verified by Sanger sequencing (Eurofins Genomics) and subsequently transformed to *E. coli* BL21 (DE3) (Novagen) for cultivation and expression as described above. Cells were lysed by sonication in native wash buffer (PBS with 15 mM imidazole, pH 7.4) and lysate supernatants were purified under native conditions with 2 mL HisPur Cobalt IMAC resin (ThermoFisher Scientific) per construct using batch method purification. After washing (native wash buffer) and elution (native elution buffer: PBS with 300 mM imidazole, pH 7.4) samples were buffer exchanged to PBS using PD-10 desalting columns (Cytiva). Absorbance was measured at 280 nm to calculate concentrations and purity was assessed by SDS-PAGE.

Binding to S trimer was assessed in two different set-ups using a Biacore T200 instrument (Cytiva). All experiments were run in the same experimental conditions as described above. In the first setup, S trimer [Wuhan-1, Protein Production Sweden Infrastructure (https://pps.gu.se/en)] was immobilized on the second flow cell of a Series S Sensor chip CM5 by amine coupling and using the manufacturer’s instructions to a final immobilization level 7857 RU. The first flow cell was used as a reference surface (amine coupling with ligand omitted). The homodimeric affibody constructs with a C-terminal His_6_-tag (Affi-AA, Affi-BB and Affi-CC) were injected over both flow cells at a concentration of 200 nM in triplicates. In the second setup, each of Affi-AA, Affi-BB and Affi-CC were separately coupled to three flow cells of a second chip by amine coupling, using the manufacturer’s instructions. The final immobilized ligand levels were 2466 RU for Affi-AA, 721RU for Affi-BB and 2084 RU for Affi-CC. 200 nM of S trimer was injected in triplicate over all three flow cells and the reference flow cell (amine coupling with ligand omitted).

Cloning, production and purification of Affi-A, Affi-B and Affi-C as homodimers with an N-terminal His_6_-tag and a C-terminal cysteine (Cys) was done as described above. Further details of the production and purification of modified affibody clones (His_6_-BB-AviTag) can be found in the Supporting Information. The production, preparative-HPLC purification and LCMS characterization of His_6_-tag free Affi-BB can be found in the Supporting Information.^46^

### 4.4 Synthesis and Characterization of Platinum Nanocatalysts (PtNCs)

PtNCs were synthesized following a modified protocol from Loynachan et al.^24^ The synthesized PtNCs were characterized by TEM,^47^ DLS, and zeta potential. Further details of PtNC synthesis and characterization can be found in the Supporting Information.

### 4.5 Preparation of PtNC Antibody Conjugates

Antibodies were purchased from Sino Biological: mAb-1 (40150-D001), mAb-2 (40150-D003), mAb-3 (40591-MM41). PtNC-antibody conjugates were prepared following a protocol modified from Loynachan et al.^24^ To a Protein LoBind sample tube (Eppendorf), 50 μL of PtNC (258 pM) was added, followed by 5 μL of HEPES (100 mM, pH 7) and 1.55 μL of detection mAb (1 mg mL^–1^, Sino Biological). The mixture was briefly vortexed and incubated for 3 h at RT under shaking (700 rpm). PtNC antibody conjugates were subsequently blocked via the addition of 50 μL of blocking solution (2 w/v % β-casein [Sigma] in Dulbecco’s PBS (DPBS, Sigma) for nanozyme-LISA and LFIA screening or 1 w/v % β-casein, 1 w/v % PVP 10 kDa [Sigma] in DPBS for optimized LFIA) and further incubation at RT for 1 h under shaking (700 rpm). Excess reagents were removed through three washing steps whereby conjugates were centrifuged at 1300 rcf for 10 min to form a pellet, the supernatant removed, and conjugates resuspended in 100 μL of wash buffer (0.2 w/v % β-casein, 0.2 v/v % Tween20 in DPBS or 1 w/v % β-casein, 1 w/v % PVP 10 kDa in DPBS, respective to blocking conditions). After the final wash step, the conjugate was resuspended in wash buffer and stored at 4 °C.

### 4.6 Preparation of PtNC Affibody Conjugates

To a Protein LoBind sample tube, 50 μL of PtNC (258 pM) was added, followed by the addition of 5 μL of HEPES (100 mM, pH 7) and 0.41 μL of detection affibody (1 mg mL^–1^). The mixture was briefly vortexed and incubated for 3 h at RT under shaking (700 rpm). PtNC affibody conjugates were subsequently blocked via the addition of 50 μL of blocking solution (2 w/v % β-casein for nanozyme-LISA and LFIA screening or 1 w/v % β-casein, 1 w/v % PVP 10 kDa in DPBS for optimized LFIA) and further incubation at RT for 1 h under shaking (700 rpm). Excess reagents were removed through three washing steps whereby conjugates were centrifuged at 1300 rcf for 10 min to form a pellet, the supernatant removed, and conjugates resuspended in 100 μL of wash buffer (0.2 w/v % β-casein, 0.2 v/v % Tween20 in DPBS or 1 w/v % β-casein, 1 w/v % PVP 10 kDa in DPBS, respective to blocking conditions). After the final wash step, the conjugate was resuspended in wash buffer and stored at 4 °C.

### 4.7 Preparation of AuNP Affibody Conjugates

To prepare 40 nm AuNP affibody conjugates, a modified AuNP antibody conjugate protocol from Richards et al. was utilized.^32^ To a glass vial, 250 μL of AuNP (40 nm, OD@530 = 1, BBI Solutions) was added, followed by 1.58 μL of detection affibody (1 mg mL^–1^) in a total of 50 μL conjugation buffer (100 mM HEPES, pH 7.6). The mixture was briefly vortexed and incubated for 3 h at RT without shaking. The AuNP-affibody conjugates were subsequently blocked via the addition of 25 μL of blocking solution (2 w/v % β-casein in 20 mM carbonate buffer pH 9.8) and further incubation at RT for 1 h without shaking. The solution was transferred to a Protein LoBind sample tube, and excess reagents were removed through three washing steps whereby conjugates were centrifuged at 5000 rcf for 10 min to form a pellet, the supernatant removed, and conjugates resuspended in 500 μL of wash buffer (20 mM HEPES + 0.05 v/v % Tween20). After the final wash step, the conjugate was resuspended in wash buffer and stored at 4 °C.

### 4.8 Nanozyme-Linked Immunosorbent Assay (Nanozyme-LISA)

To the appropriate wells of a 96-well clear flat bottom polystyrene high bind microplate (Corning), 100 μL of capture immunoreagent (mAb or affibody, 1 μg mL^–1^) was added and incubated overnight at 4 °C. The plate was subsequently washed (3x) with DPBST (DPSB + 0.05 v/v % Tween20). All wells were blocked through the addition of 150 μL of 3 w/v % BSA in DPBS and incubated at RT for 1 h. The wells were subsequently washed (3x) with DPBST. 100 μL of antigen-spiked PBST (SARS-CoV-2 S protein, varying concentrations) was then added to the appropriate wells and incubated at RT for 30 min, before wells were then washed (3×) with DPBST. 100 μL of PtNC conjugate (mAb of affibody conjugate, 2 pM) was added to the appropriate wells and incubated at RT for 30 min. The wells were washed (3×) with DPBST before the addition of 100 μL of freshly prepared TMB solution (1.6 v/v % TMB (0.6 mg mL^–1^) [Sigma] in DMSO [Sigma] + 0.4 v/v % 5% H_2_O_2_ [Sigma] in 50 mM citrate buffer, pH 5.0). The plate was shielded from light and incubated at RT for 30 min. After exactly 30 min, the reaction was stopped through the addition of 50 μL of H_2_SO_4_ (4 M) to each well. The OD at 450 nm was recorded using a SpectraMax M5 microplate reader (Molecular Devices) and analyzed using GraphPad Prism and Detection Limit Fitting Tool.^48−50^

### 4.9 Lateral Flow Immunoassay Printing and Fabrication

To produce lateral flow strips with specific capture affibodies and antibodies, an automated liquid dispensing system (BioDot System AD3220) was used. Here, all capture immunoreagents (mAb or affibody, 1 mg mL^–1^) were filtered through a 0.22 μm filter. The capture immunoreagent was dispensed at a height of 10 mm from the base of the nitrocellulose membrane (CN95 Unisart Nitrocellulose Membrane, Sartorius) before drying for 4 h at 37 °C. Half-dipstick lateral flow strips were assembled onto backing card (KN-PS1060.18, Kenosha) with an overlapping absorbent pad (KN-222-20.1, Ahlstrom-munksjo). Half-dipstick strips were then cut into individual strips with a width of 3 mm using an automated guillotine (BioDot CM5000 Guillotine Cutting System). Half-dipstick strips were stored in a desiccant cabinet before use, unless otherwise stated.

### 4.10 PtNC Lateral Flow Immunoassay

All PtNC lateral flow immunoassays were performed by immersing a constructed LFIA test strip (mAb or affibody capture) into a 96-well clear flat bottom polystyrene nonbinding surface microplate (Corning) containing. Each well contained: 50 μL sample (S protein spiked into DPBST), 15 μL of PtNC conjugate (mAb or affibody conjugate, 100 pM). When the solution had fully wicked up the LFIA strip (ca. 10 min), the strip was transferred into a well containing 100 μL of running buffer (0.2 w/v % β-casein, 0.2 v/v % Tween20 in DPBS) for 10 min. Next, the strip was transferred to an amber Eppendorf containing 500 μL of amplification solution (Pierce CN/DAB (4-chloro-1-naphthol/3,3′-diaminobenzidine, tetrahydrochloride) Substrate Kit [ThermoFisher Scientific], adjusted with hydrogen peroxide solution 30 w/w % [Sigma] to a final added peroxide concentration of 4 M) for 10 min. Finally, the strip was moved into an Eppendorf containing 1 mL of Milli-Q water and briefly agitated to stop the reaction. Strips were left to dry for 20 min before being photographed with an iPhone 13 camera and images processed using ImageJ. Extracted pixel intensities were analyzed using GraphPad Prism and Detection Limit Fitting Tool.^48−50^

### 4.11 AuNP Lateral Flow Immunoassay

All AuNP lateral flow immunoassays were performed by immersing a constructed affibody LFIA test strip into a 96-well clear flat bottom polystyrene nonbinding surface microplate. Each well contained: 50 μL sample (S protein spiked into running buffer (0.2 w/v % β-casein, 0.2 v/v % Tween20 in DPBS), fetal bovine serum [Sigma] + 0.05 v/v % Tween20 or 3:2 human pooled saliva [Lee BioSolutions]: running buffer), 20 μL of AuNP affibody conjugate (OD@530 = 1). When the solution had fully wicked up the LFIA strip (ca. 15 min), the strip was transferred into a well containing 100 μL of running buffer (0.2 w/v % β-casein, 0.2 v/v % Tween20 in DPBS) for 10 min. LFIA strips were images with an iPhone 13 camera and images processed using ImageJ. Extracted pixel intensities were analyzed using GraphPad Prism and Detection Limit Fitting Tool.^48−50^

### 4.12 AuNP Aging Study

All AuNP lateral flow immunoassays were performed as described above, using running buffer (0.2 w/v % β-casein, 0.2 v/v % Tween20 in DPBS) as a sample matrix, with a final concentration of S protein of 2 nM. LFIA test strips were stored under a range of conditions to assess the stability of the capture protein. The storage conditions were desiccator storage (standard storage conditions, 20.2 ± 0.77 °C, 22.7 ± 3.0% humidity), ambient conditions (21.8 ± 0.44 °C, 58.0 ± 4.60% humidity), a humid environment (21.4 ± 0.48 °C, 78.8 ± 1.70% humidity), and elevated temperatures (45.3 ± 0.34 °C, 22.4 ± 2.26% humidity).

### 4.13 ImageJ Processing

Images of LFIA strips were captured using an iPhone 13 camera. Images imported into ImageJ (ImageJ 1.53k) and converted to gray scale (8 bit) images. A region of interest around the test line was selected (width to height ratio 87:216), and peak area pixel density was calculated using the ImageJ software. Test line intensities were normalized to a reference point (grid line) in the image to account for any variation in image lighting.

### 4.14 Brief Computational Details

All MD simulations were performed in triplicate using GROMACS 2018.3.^51,52^ Homology modeling, as implemented in MODELLER 10.4 was used to build all dimeric affibody constructs, while the structure for the RBD of SARS-CoV-2 S protein was sourced from the Protein Data Bank (PDB ID: 7R8L).^53,54^ The CHARMM27^55^ force field, was used to model the affibodies in solution and protein–protein complexes; the GolP-CHARMM^56^ force field, employed to characterize the intermolecular interaction between the affibody and the surface of Au(111); and the CHARMM compatible *ab initio* derived force field was utilized to model the citrate anions.^57^ Simulations for the protein–surface systems and affibody dimers free in solution were run for 500 ns, with timesteps of 1 and 2 fs, respectively. The affibody–antigen protein complexes were simulated for 200 ns simulations with a time step of 2 fs. Please refer to the Supporting Information for more detailed computational methodology.

### 4.15 Simulation Data Analysis

Visual Molecular Dynamics (VMD) 1.9.3 was used to visualize and analyze the final combined 150 ns of equilibrated trajectories.^58^ Cluster analysis (cutoff value of 0.25 nm) was used to obtain representative median structures of Affi-AA and Affi-BB in aqueous solutions.^59^ Principal Component Analysis (PCA) was applied to identify concerted motions of the dimeric affibodies adsorbed on the Au(111) surface, as described in an earlier study.^60^ The robustness of each principal component was evaluated using the cosine value calculation where low values indicate essential motions and high values indicate random diffusion of molecules.^61^ To quantify the Gibbs free energy of protein–protein complexes, MM-PBSA was computed as previously described,^62,63^ with a value of 20 for the solute dielectric constant.^64^ Calculations were performed on the last equilibrated 10 ns, with 2000 bootstrap iterations.^65^ Hydrogen bonding analysis, in the affibody-RBD complexes was also performed on the last 10 ns of the equilibrated trajectories.

## Supporting Information Available

Characterization of affibody clones; characterization of PtNC and AuNPs; screening of affinity agents in plate-based assays; optimization of LFIAs; serial dilution LFIA images; stability study LFIA images; purification and characterization of His_6_-tag free affibody; additional experimental methods including affibody production and purification, synthesis of PtNCs, characterization of nanoparticles using DLS, zeta potential and TEM, statistical analysis; additional details of computational analysis using molecular dynamics simulations, including model construction, simulation set up and parameters, and additional computational results (PDF)
