# Supplementary material for: Adding a Twist to Lateral Flow Immunoassays: A Direct Replacement of Antibodies with Helical Affibodies, from Selection to Application
Source: J Am Chem Soc. 2025 Mar 26;147(14):11925–40. doi: 10.1021/jacs.4c17452 (PMC11987028; doi:10.1021/jacs.4c17452)
Supplement: Supplementary file 1 — ja4c17452_si_001.pdf [file ja4c17452_si_001.pdf]

Supporting Information

Adding a twist to lateral flow immunoassays: a direct replacement of antibodies with helical affibodies, from selection to application

Authors:

Christy J. Sadler[1,2†], Adam Creamer[1,2†], Kim Anh Giang[3], Kevion K. Darmawan[4], André Shamsabadi[1,2], Daniel A. Richards[1,5], Johan Nilvebrant[3], Jonathan P. Wojciechowski[1,2], Patrick Charchar[4], Ross Burdis[1], Francesca Smith[1], Irene Yarovsky[4], Per-Åke Nygren[3], Molly M. Stevens[1,2*]

Affiliations:

[1] Department of Materials, Department of Bioengineering, Institute of Biomedical Engineering Imperial College London, London, SW7 2AZ, UK

[2]Department of Physiology, Anatomy and Genetics, Department of Engineering Science, Kavli Institute for Nanoscience Discovery, University of Oxford, OX1 3QU, UK

[3] Department of Protein Science, AlbaNova University Center, KTH Royal Institute of Technology, SE-114 21 Stockholm, Sweden

[4] School of Engineering, RMIT University, Melbourne, VIC, 3001, Australia

[5] Institute for Chemical and Bioengineering, ETH Zurich, 8093 Zürich, Switzerland

[†]These authors contributed equally

*Corresponding author (molly.stevens@dpag.ox.ac.uk)

Contents:

Supporting Information Figures: *p02*

Supporting Information Experimental Section: *p25*

Supporting Information Computational Details: *p30*

References: *p39*

Supporting Information Figures

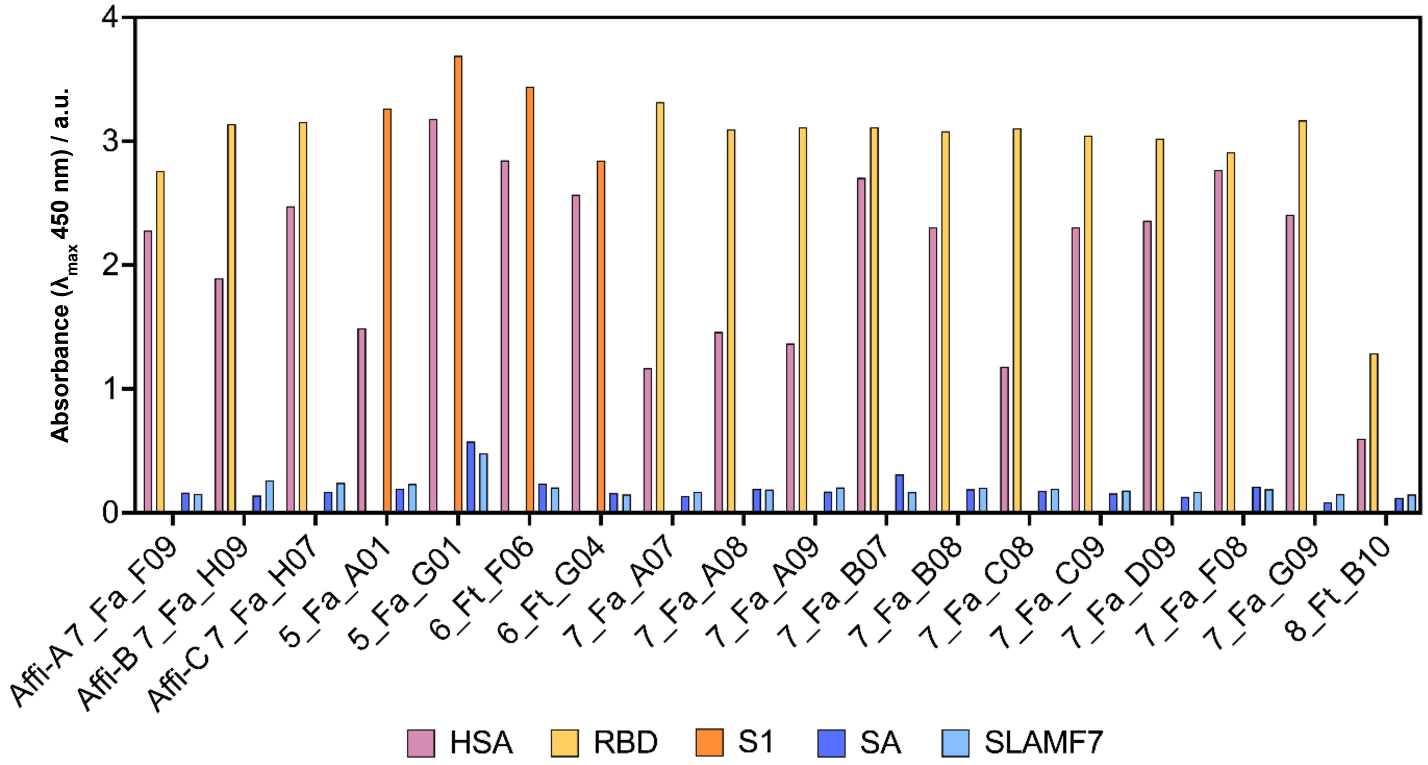

Figure S1: Monoclonal phage ELISA absorbance values of 18 affibody constructs, displayed as fusion proteins with an albumin binding domain (ABD) on the phage surface. The ABD is used as a positive control for affibody display when using human serum albumin (HSA) as a target. The affinity towards the SARS-CoV-2 S1 protein and receptor-binding domain (RBD) are used to assess the ability of the affibody clones to specifically bind to the target antigen. Streptavidin (SA) and surface antigen CD319 (SLAMF7) are utilized as negative controls, n = 1.

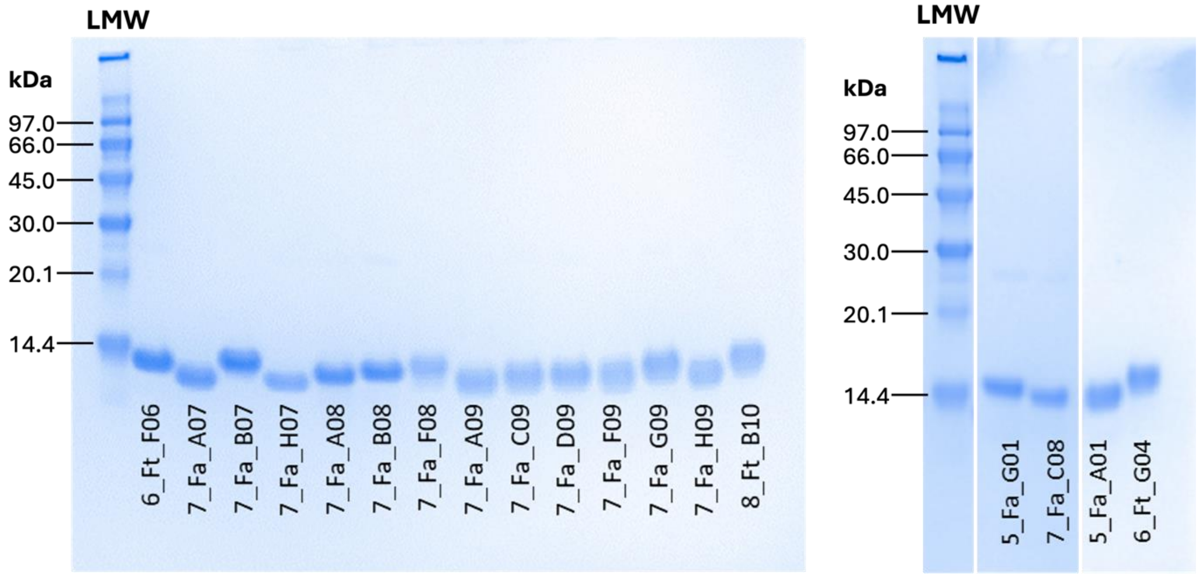

Figure S2: SDS-PAGE gel of monomeric affibody constructs with albumin binding domain (ABD) fusion partner, compared to the low molecular weight (LMW) ladder.

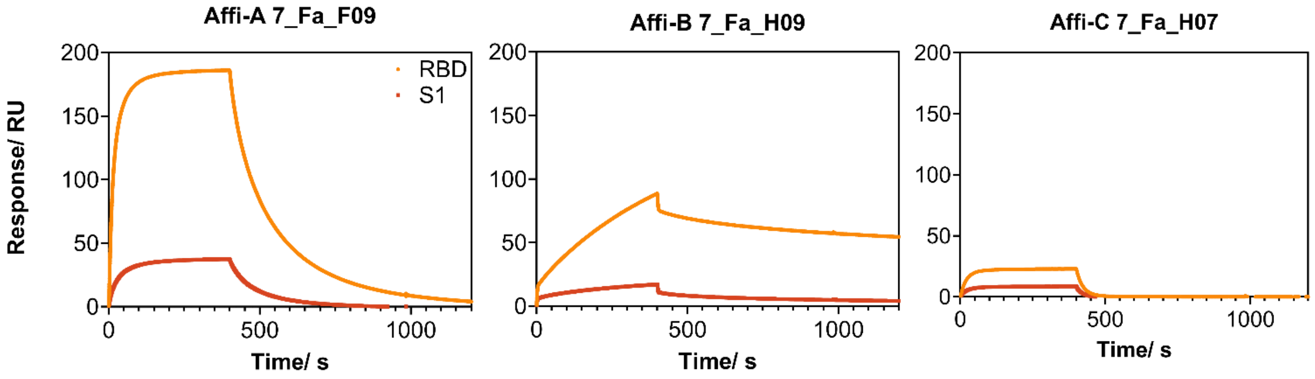

Figure S3: Surface plasmon resonance (SPR) sensorgrams for Affi-A, Affi-B, and Affi-C constructs. The RBD (light orange line) or S1 (dark orange line) antigens are immobilized onto the sensor surface, and the monomeric affibody constructs are injected over the sensor, n = 1.

			Helix 1		Helix 2		Helix 3		
	**Library**	VDNKFNKE	XXXAXXEIXX	LPNLNX	XQXXAFXXSLXD	DPSQ	SANLLAEAKKLND	AQAPK	
**Affi-A**	**7_Fa_F09**	--------	IMW-WG--LK	-----F	E-KG--YK--W-	----	-------------	-----	x6
**Affi-B**	**7_Fa_H09**	--------	YGN-MS--RY	-----N	E-RE--IT--F-	----	-------------	-----	x1
**Affi-C**	**7_Fa_H07**	--------	WDD-KH--RM	-----N	A-IE--IA--W-	----	-------------	-----	x1
	**5_Fa_A01**	--------	YIM-EN--FW	-----I	T-KR--IN--W-	----	-------------	-----	x37
	**5_Fa_G01**	--------	EWR-YH--KE	-----K	W-QR--YA--H-	----	-------------	-----	x1
	**6_Ft_F06**	--------	HED-YR--RA	-----I	W-SR--IN--Y-	----	-------------	-----	x1
	**6_Ft_G04**	--------	HQK-YH--DE	-----I	W-KR--KV--F-	----	-------------	-----	x1
	**7_Fa_A07**	--------	ILR-NA--WA	-----V	A-IH--ID--Y-	----	-------------	-----	x1
	**7_Fa_A08**	--------	HKA-WS--ME	-----I	W-KR--II--G-	----	-------------	-----	x1
	**7_Fa_A09**	--------	YIW-EH--FE	-----L	M-RR--IG--W-	----	-------------	-----	x1
	**7_Fa_B07**	--------	IKQ-LR--NE	-----R	L-RL--HN--S-	----	N------------	-----	x1
	**7_Fa_B08**	--------	HLL-HR--SE	-----I	W-KR--YT--H-	----	-------------	-----	x2
	**7_Fa_C08**	--------	WRN-YN--YA	-----I	W-KR--YN--F-	----	-------------	-----	x5
	**7_Fa_C09**	--------	AMQ-HK--EG	-----V	W-RR--YI--F-	----	-------------	-----	x2
	**7_Fa_D09**	--------	ERK-YS--QQ	-----I	W-KR--YA--F-	----	-------------	-----	x2
	**7_Fa_F08**	--------	IEQ-HG--RN	-----E	L-RI--HY--E-	----	-------------	-----	x1
	**7_Fa_G09**	--------	HHK-YN--NA	-----E	W-KR--YA--F-	----	-------------	-----	x1
	**8_Ft_B10**	--------	IQQ-GI--DK	-----S	D-NI--HQ--R-	----	-------------	-----	x4

Figure S4: Amino acid sequence alignment for the affibodies candidates compared to the library gene. Positions marked with 'X' in the library gene correspond to the positions subjected to combinatorial randomization in the library. The multiplication factor indicates the number of times the candidates appeared in the sequence analysis of positive clones from the post-selection monoclonal phage ELISA screening.

Table S1: Table of DLS and Zeta potential parameters for PtNCs before and after conjugation with mAb and affibody affinity agents. Data given as mean ± standard deviation, n = 3.

Sample	Detection Protein	Z-Average/ nm	PDI	Intensity Mean/ nm	Volume Mean/ nm	Number Mean/ nm	Zeta Potential/ mV
Bare PtNC	N/A	112.0 ± 1.058	0.012 ± 0.005	116.5 ± 0.6083	114.8 ± 1.277	96.26 ± 2.225	-46.3 ± 1.07
PtNC Affibody	Affi-BB-Cys	133.3 ± 1.375	0.081 ± 0.021	147.1 ± 2.848	159.6 ± 4.75	104.5 ± 9.40	-12.5 ± 1.29
PtNC mAb	mAb-3	129.9 ± 0.9019	0.056 ± 0.005	138.7 ± 1.274	147.1 ± 1.966	114.2 ± 1.277	-14.8 ± 1.05

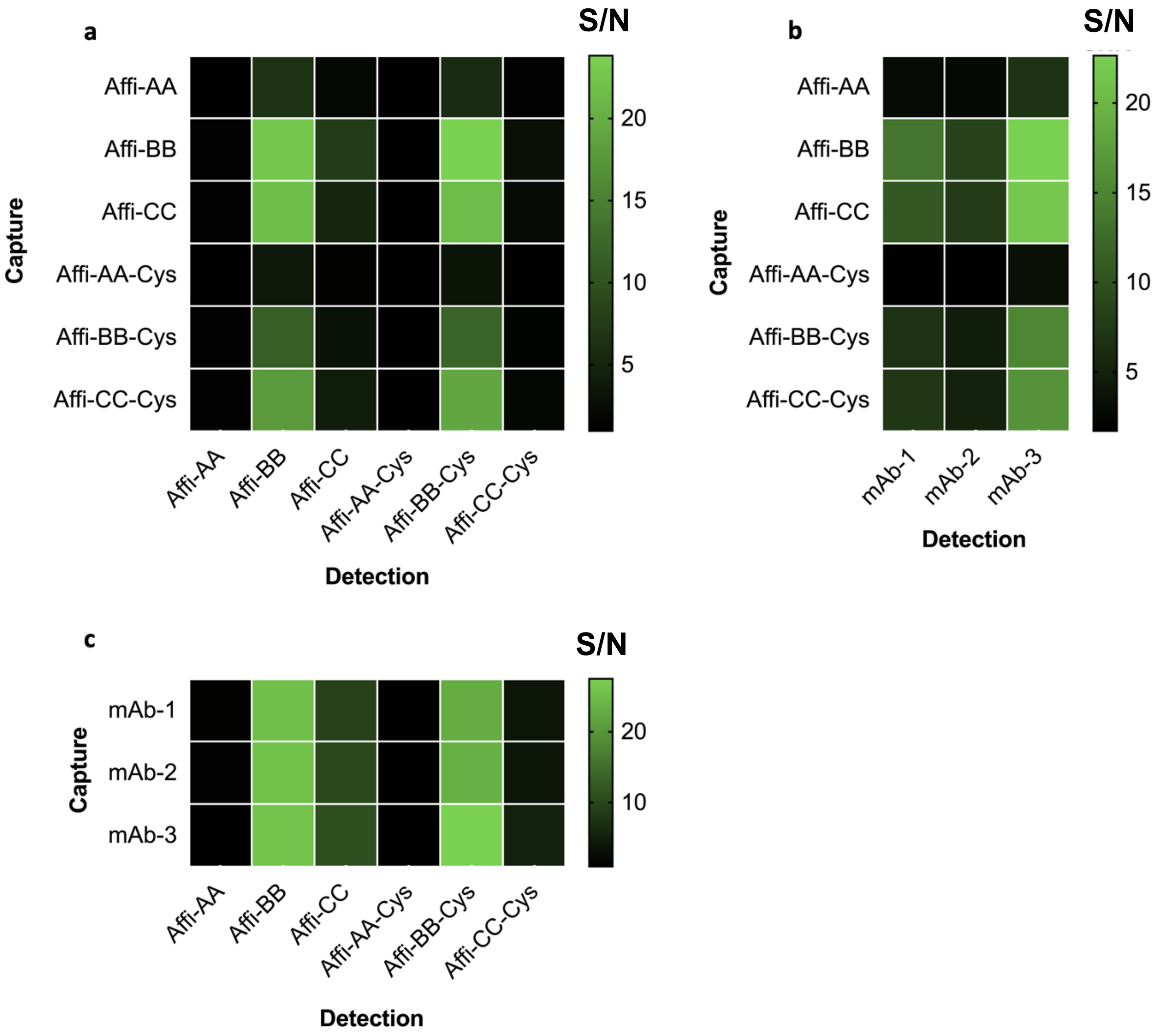

Figure S5: Heatmap illustrating the signal-to-noise (S/N) ratio extracted from nanozyme-LISAs. a) Utilizing affibody proteins as capture and PtNCs as detection probes, n = 2. b) Utilizing affibody proteins as capture and mAb PtNCs as detection probes, n = 2. c) Utilizing mAbs as capture and affibody PtNCs as detection probes, n = 2.

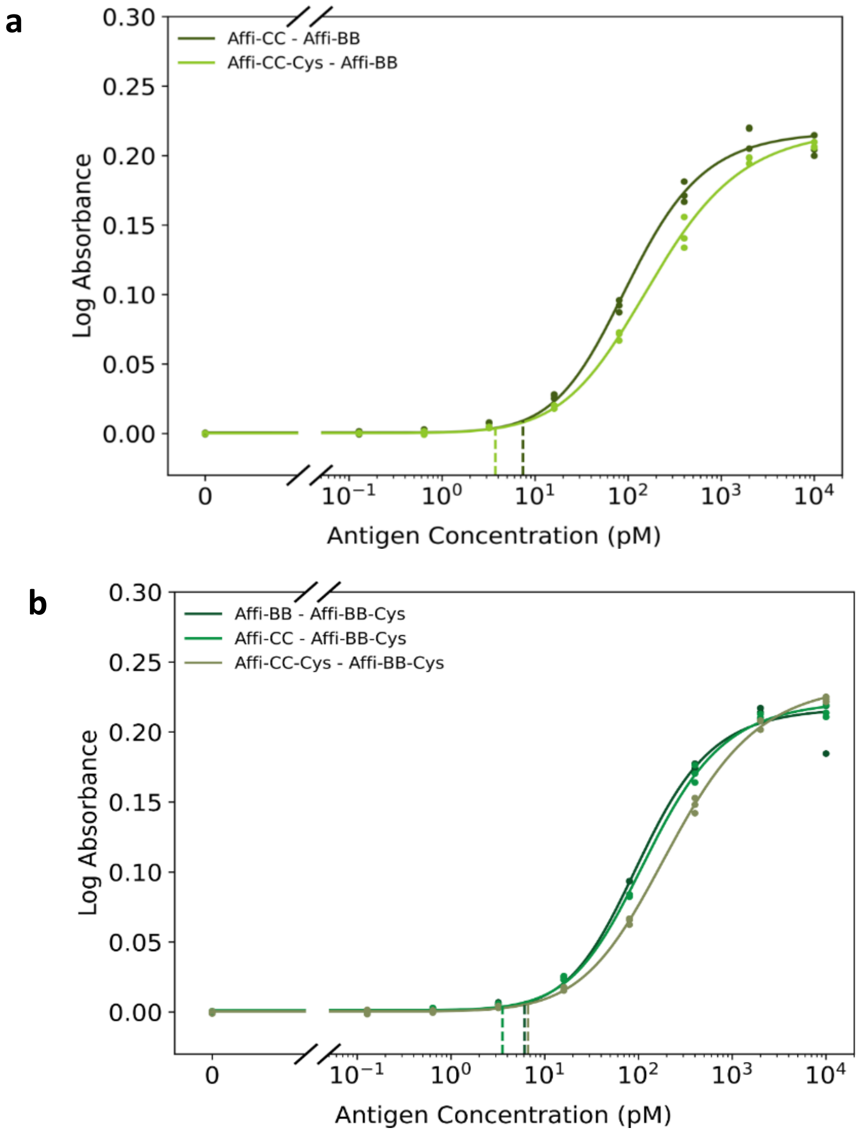

Figure S6: Serial dilution of S trimer antigen in nanozyme-LISA format to assess the ability of capture affibodies to function with various PtNC detection probes, where the dashed line represents the antigen concentration at the LOD. a) Nanozyme-LISA utilizing PtNC Affi-BB detection probes, n = 3. b) Nanozyme-LISA utilizing PtNC Affi-BB-Cys detection probes, n = 3.

Table S2: Output from 4-parameter logistic regression limit of detection fitting of serial dilution of S trimer antigen in nanozyme-LISA format using affibody proteins as capture and detection probes.

Capture	Detection	LC	LD	LOD/ pM	LOD Lower/ pM	LOD Upper/ pM	RMSE in logY domain
Affi-BB	Affi-BB	0.0025	0.0041	2.44	0.86	6.88	0.0039
Affi-BB	Affi-BB-Cys	0.0043	0.0063	6.08	2.24	16.52	0.0065
Affi-CC	Affi-BB	0.0016	0.0092	7.38	3.95	13.78	0.0057
Affi-CC	Affi-BB-Cys	0.0020	0.0039	3.54	1.31	9.57	0.0032
Affi-CC-Cys	Affi-BB	0.00074	0.0036	3.74	1.32	10.55	0.0037
Affi-CC-Cys	Affi-BB-Cys	0.0027	0.0058	6.63	4.40	10.01	0.0023

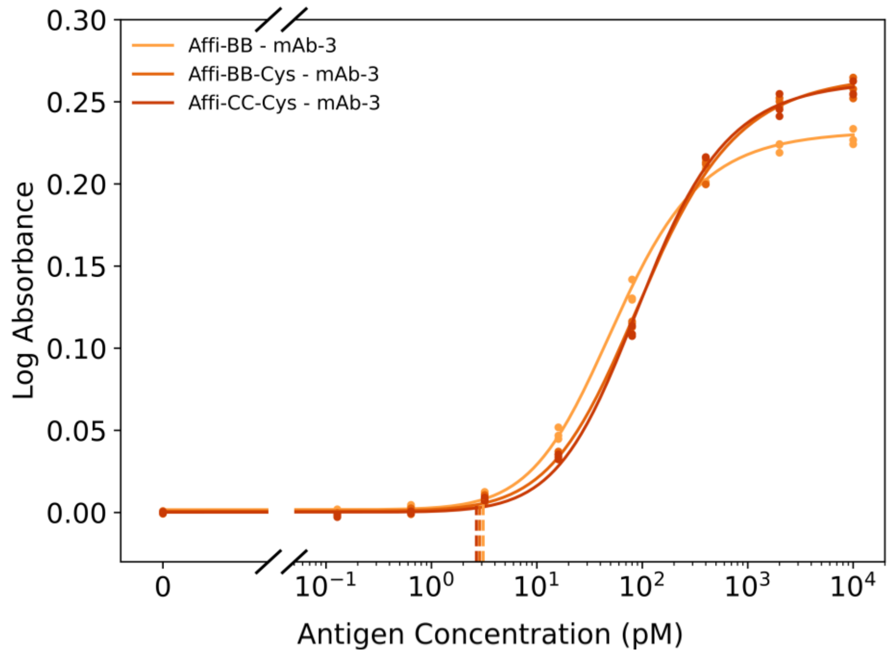

Figure S7: Serial dilution of S trimer antigen in nanozyme-LISA format to assess the ability of capture affibodies to function with PtNC mAb detection probes, where the dashed line represents the antigen concentration at the LOD, n = 3.

Table S3: Output from 4-parameter logistic regression limit of detection fitting of serial dilution of S trimer antigen in nanozyme-LISA format using affibody proteins as capture and mAb detection probes.

**Capture**	**Detection**	**LC**	**LD**	**LOD/ pM**	**LOD Lower/ pM**	**LOD Upper/ pM**	**RMSE in logY domain**
Affi-BB	mAb-3	0.0018	0.0080	3.10	1.75	5.49	0.0041
Affi-CC	mAb-3	0.0020	0.0045	2.52	0.95	6.68	0.0047
Affi-BB-Cys	mAb-3	0.0024	0.0051	2.87	1.43	5.74	0.0033
Affi-CC-Cys	mAb-3	0.0013	0.0027	2.67	0.73	9.70	0.0042

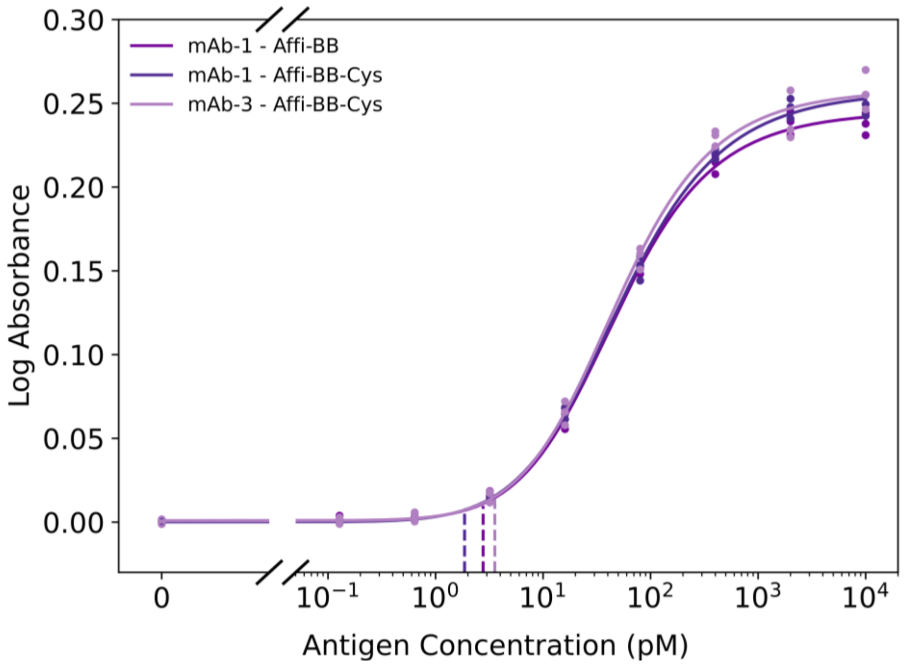

Figure S8: Serial dilution of S trimer antigen in nanozyme-LISA format to assess the ability of capture mAbs to function with PtNC affibody detection probes, where the dashed line represents the antigen concentration at the LOD, n = 3.

Table S4: Output from 4-parameter logistic regression limit of detection fitting of serial dilution of S trimer antigen in nanozyme-LISA format using mAb proteins as capture and affibody detection probes.

Capture	Detection	LC	LD	LOD/ pM	LOD Lower/ pM	LOD Upper/ pM	RMSE in logY domain
mAb-1	Affi-BB	0.0039	0.011	2.77	1.83	4.19	0.0041
mAb-1	Affi-BB-Cys	0.00086	0.0073	1.86	1.13	3.06	0.0036
mAb-3	Affi-BB	0.0016	0.0059	1.85	0.99	3.47	0.0039
mAb-3	Affi-BB-Cys	0.0034	0.015	3.56	2.19	5.78	0.0064

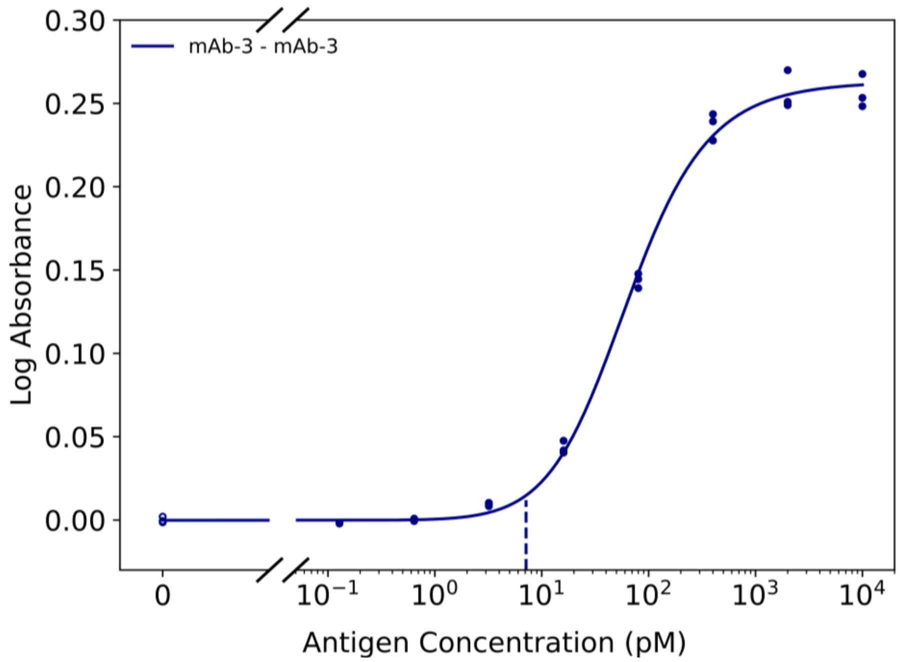

Figure S9: Serial dilution of S trimer antigen in nanozyme-LISA format to assess the ability of capture mAbs to function with PtNC mAb detection probes, where the dashed line represents the antigen concentration at the LOD, n = 3.

Table S5: Output from 4-parameter logistic regression limit of detection fitting of serial dilution of S trimer antigen in nanozyme-LISA format using mAb proteins as capture and mAb detection probes.

Capture	Detection	LC	LD	LOD/ pM	LOD Lower/ pM	LOD Upper/ pM	RMSE in logY domain
mAb-1	mAb-3	0.0030	0.014	2.24	1.45	3.48	0.0059
mAb-3	mAb-3	0.0052	0.015	7.13	4.91	10.36	0.0058

Table S6: Table comparing the T-test statistical significance of calculated LOD values for the top performing affinity agent pairs in nanozyme-LISA serial dilution experiments.

Dataset 1		Dataset 2		LOD 1/ pM	LOD 2/ pM	P-Value
**Capture**	**Detection**	**Capture**	**Detection**			
mAb-1	mAb-3	Affi-BB	Affi-BB	2.24	2.44	0.886
mAb-1	mAb-3	Affi-CC	mAb-3	2.24	2.52	0.831
mAb-1	mAb-3	mAb-3	Affi-BB	2.24	1.85	0.627

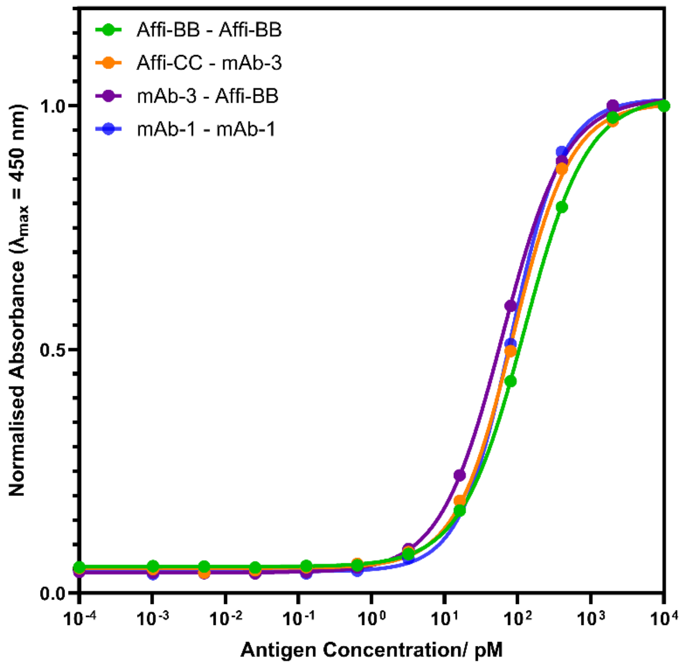

Figure S10: Normalized sigmoidal curves of S protein dilution with the four combinations of antibody and affibody as capture and/or detection probes, where the dashed line represents the antigen concentration at the LOD, n = 3.

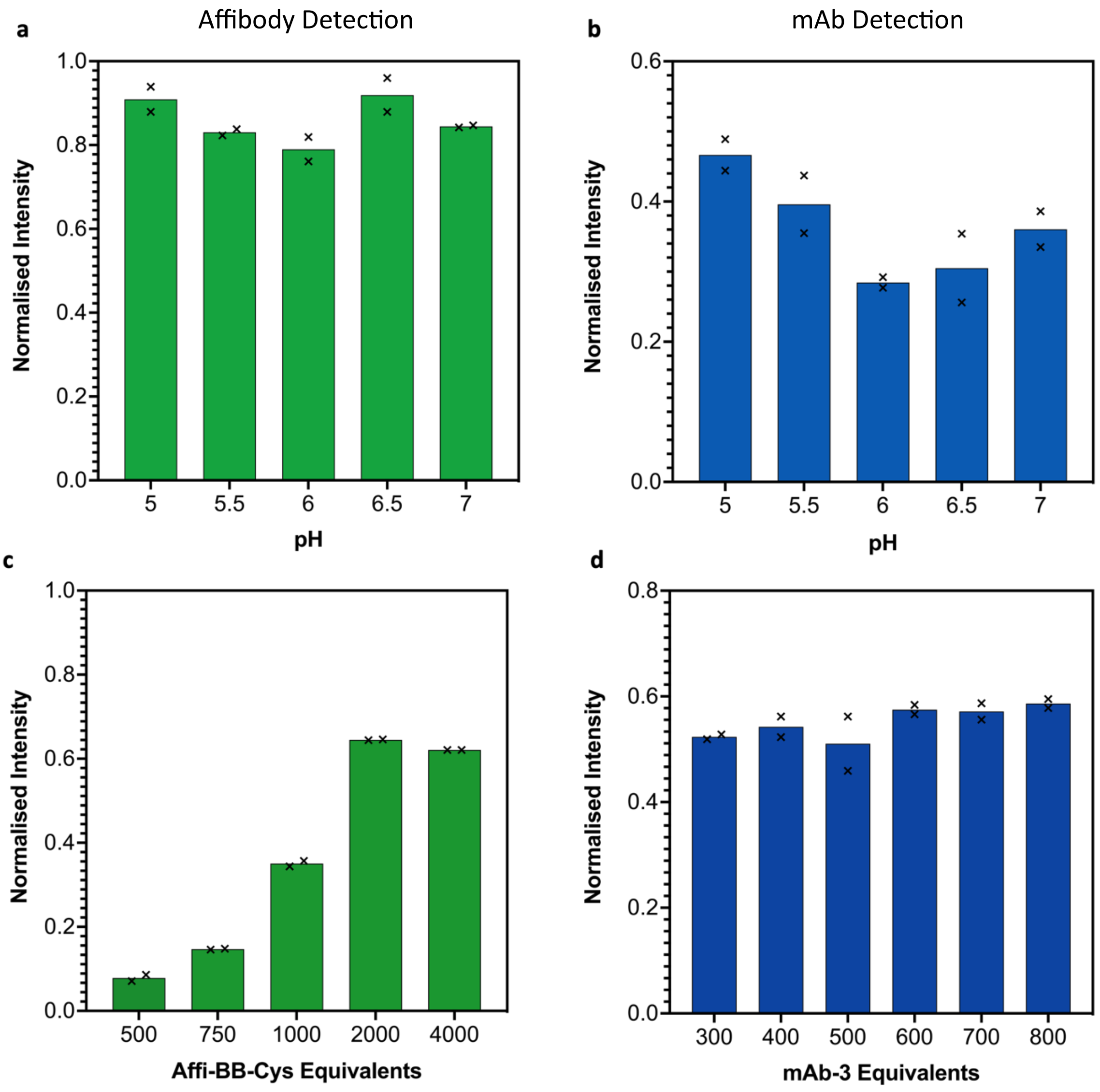

Figure S11: Screening of conjugation conditions to produce PtNC affinity agent conjugates. Screening was performed in half dipstick LFIA format, assessing the test line intensity with and without the presence of S trimer antigen (400 and 0 pM, respectively), n = 2. a) Screening of pH of affibody PtNC conjugation protocol. LFIA performed utilizing Affi-BB-Cys capture probe and test line intensity assessed prior to catalytic amplification. b) Screening of pH of mAb PtNC conjugation protocol. LFIA performed utilizing Affi-BB capture probe and test line intensity assessed after catalytic amplification. c) Screening of number of affibody to PtNC equivalents. LFIA performed utilizing Affi-BB-Cys capture probe and test line intensity assessed prior to catalytic amplification. d) Screening of number of mAb-3 to PtNC equivalents. LFIA performed utilizing Affi-BB capture probe and test line intensity assessed after catalytic amplification.

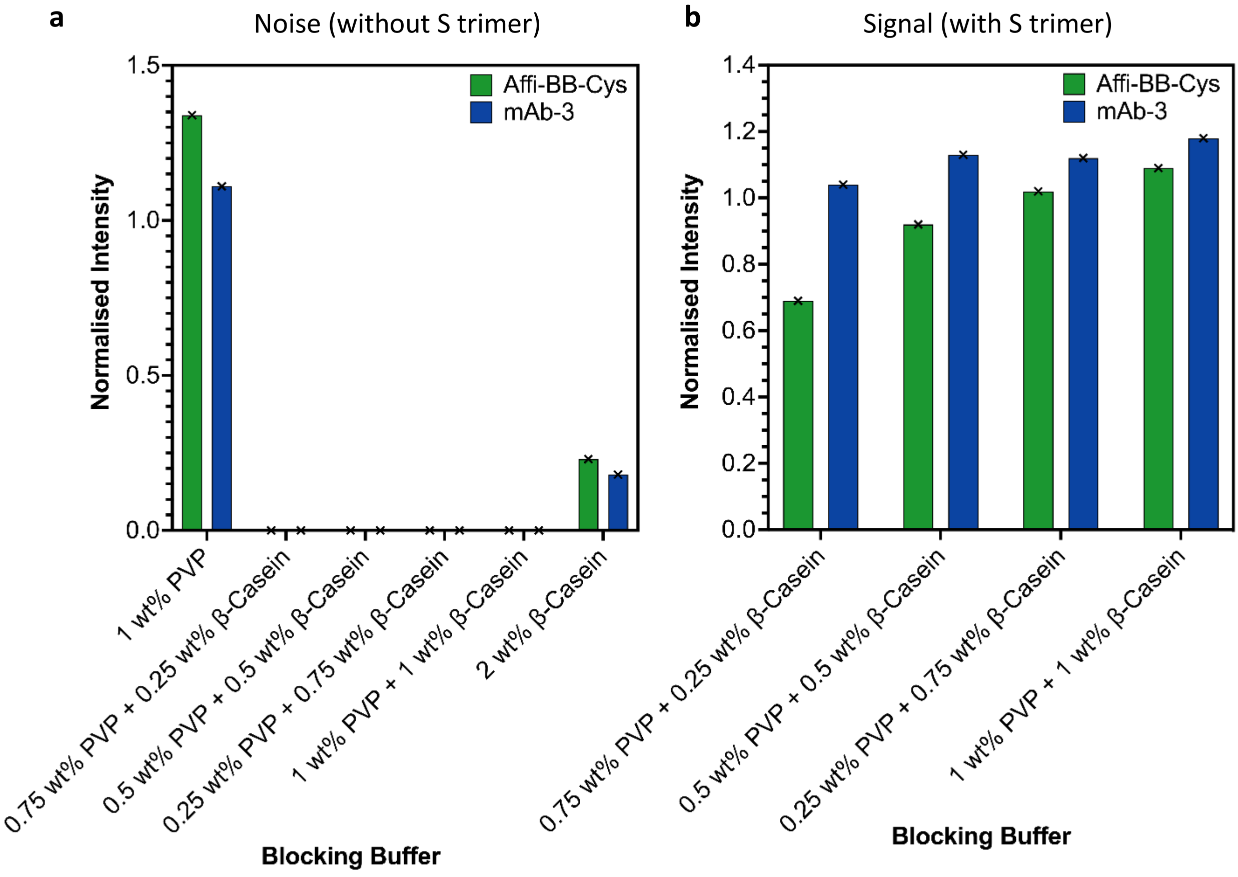

Figure S12: Screening of conjugation blocking buffer using affibody and mAb PtNC detection probes and Affi-BB-Cys capture probe. Screening was performed by assessing the test line intensity with and without the presence of S trimer antigen (400 and 0 pM, respectively). a) Test line intensity without antigen (noise) for all blocking conditions after PtNC amplification, n = 2. b) Test line intensity with antigen (signal) for all blocking conditions after PtNC amplification, n = 1.

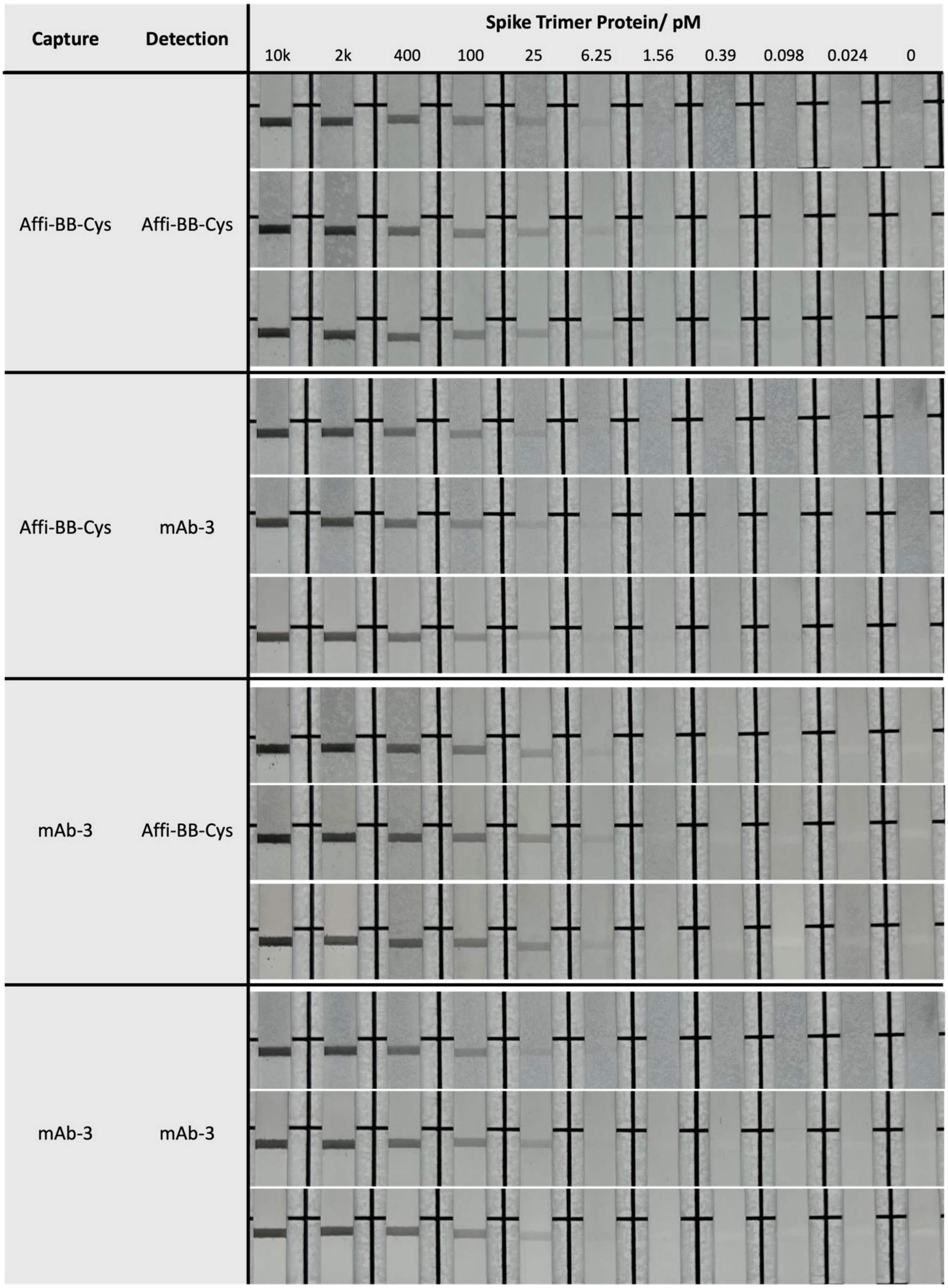

Figure S13: LFIA strips utilizing a serial dilution of S trimer antigen with affibody and mAb capture and detection probes. Photographs represent pre-processed images of three repeats.

Table S7: Table comparing the T-test statistical significance of calculated LOD values for PtNC LFIA serial dilution experiments.

**Dataset 1**		**Dataset 2**		**LOD 1/ pM**	**LOD 2/ pM**	**P-Value**
**Capture**	**Detection**	**Capture**	**Detection**			
mAb-3	mAb-3	Affi-BB-Cys	Affi-BB-Cys	5.60	3.33	0.224
mAb-3	mAb-3	mAb-3	Affi-BB-Cys	5.60	2.11	0.042
mAb-3	mAb-3	Affi-BB-Cys	mAb-3	5.60	11.42	0.060

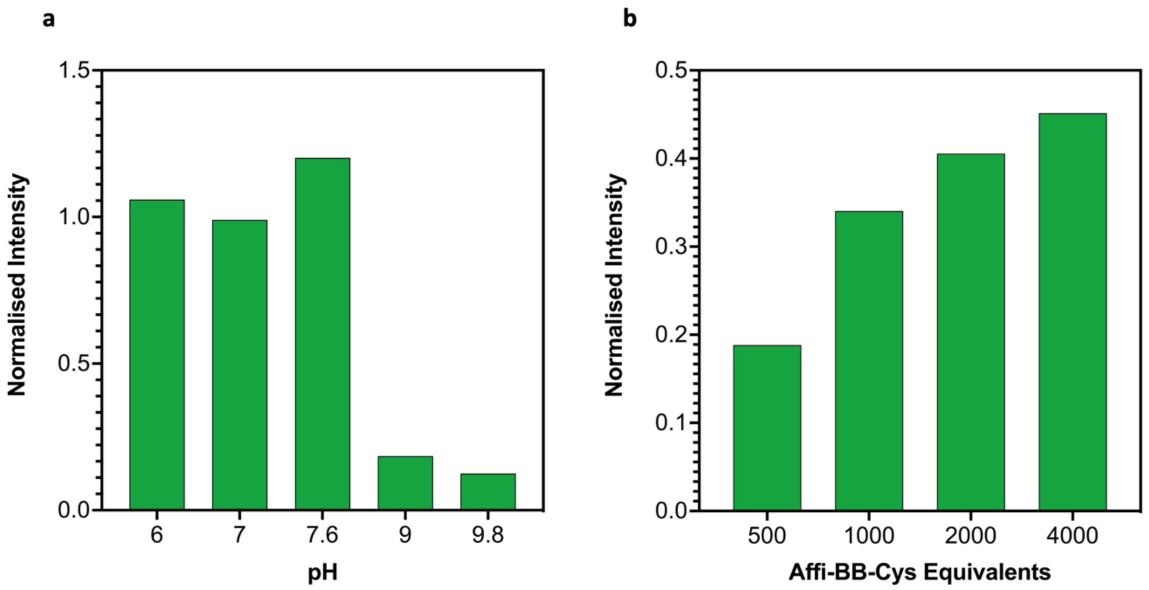

Figure S14: Screening of conditions for AuNP affibody conjugation optimization. Screening was performed by assessing the test line intensity with and without the presence of S trimer antigen. a) Test line intensity with variation of conjugation pH utilizing Affi-BB-Cys capture. Signal generated using 10 nM S trimer. No signal without antigen was observed, n = 1. b) Test line intensity with variation of affibody to AuNP equivalents utilizing Affi-BB-Cys capture. Signal generated using 400 pM S trimer. No signal without antigen was observed, n = 1.

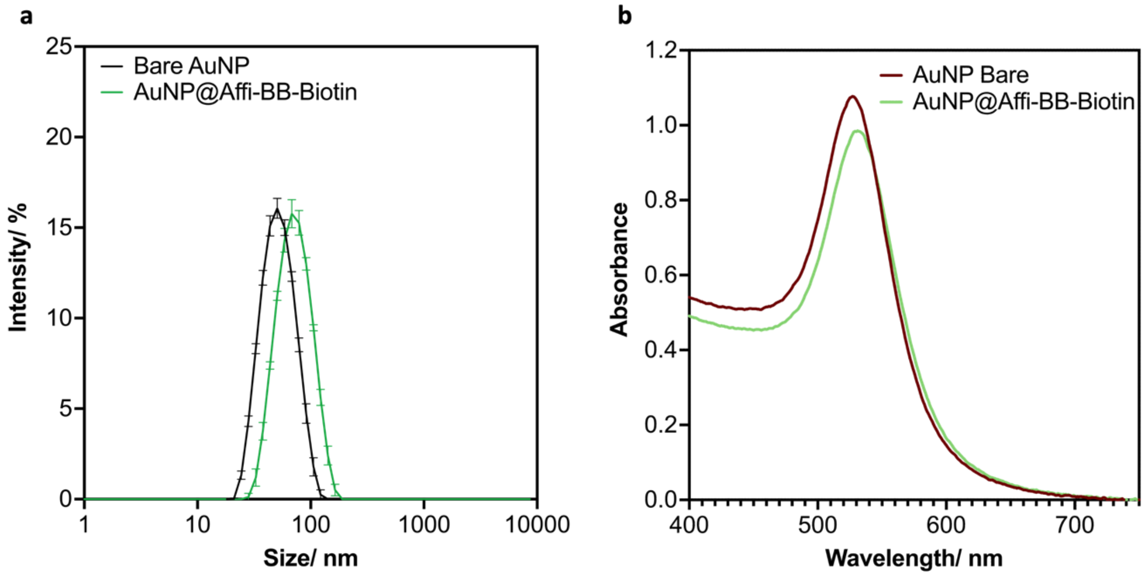

Figure S15: a) Dynamic light scattering measurements showing the intensity distribution of AuNP size before (black) and after conjugation with affibody affinity agents (green), n = 3. b) UV-Vis spectra of 40 nm AuNP and AuNP@affibody conjugates.

Table S8: Table of DLS and Zeta potential parameters for AuNPs before and after conjugation with affibody affinity agents. Data given as mean ± standard deviation, n = 3.

**Sample**	**Detection Protein**	**Z-Average/ nm**	**PDI**	**Intensity Mean / nm**	**Volume Mean / nm**	**Number Mean / nm**	**Zeta Potential/ mV**
Bare AuNP	N/A	46.00 ± 0.261	0.159 ± 0.008	54.1 ± 0.8028	38.45 ± 0.4965	31.82 ± 0.5860	-49.6 ± 1.69
AuNP Affibody	Affi-BB-Biotin	65.28 ± 0.5479	0.143 ± 0.008	74.93 ± 0.6198	52.47 ± 1.591	42.24 ± 2.15	-32 ± 0.651

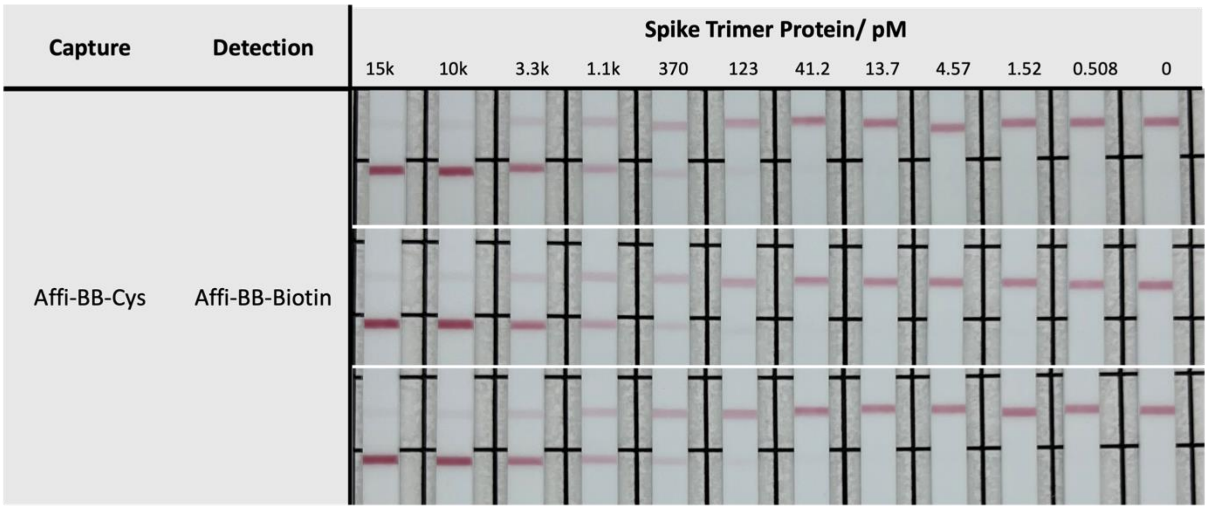

Figure S16: LFIA strips utilizing a serial dilution of S trimer antigen with affibody (Affi-BB-Cys) capture probes, polystreptavidin R control line and AuNP Affi-BB-Biotin detection probes. Pre-processed photographs of three repeats.

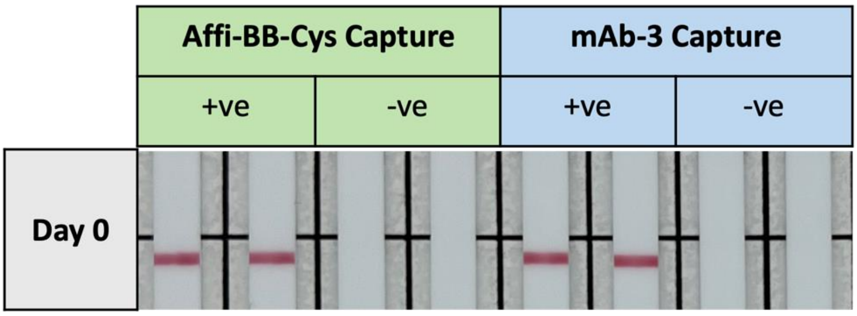

Figure S17: Images of half dipstick LFIA strips utilizing Affi-BB-Cys and mAb-3 capture probes alongside AuNP Affi-BB-Cys detection probes. The strips represent newly fabricated half dipstick LFIAs. +ve represents the signal at 10 nM and -ve represents the noise utilizing 0 nM Spike trimer, n = 2.

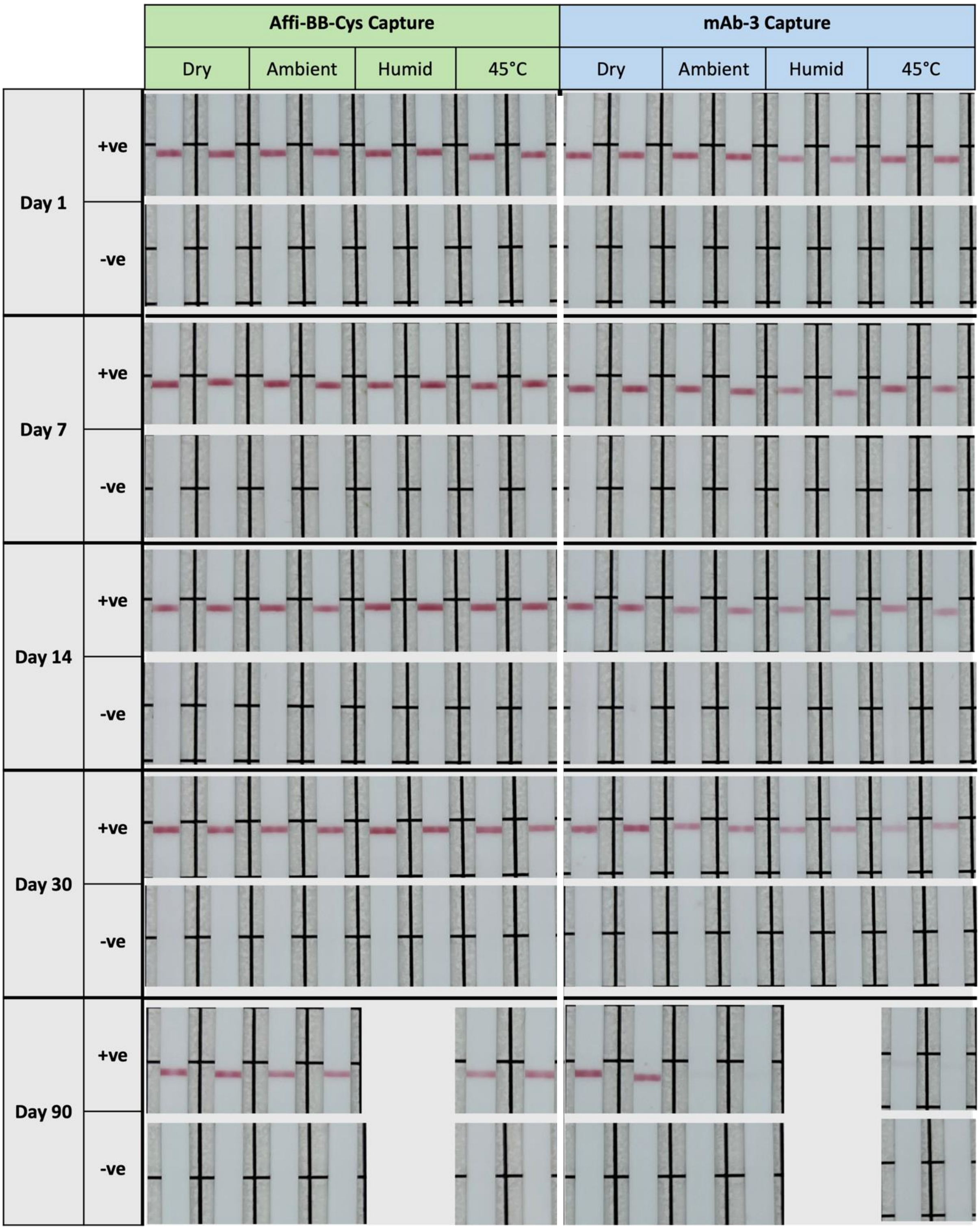

Figure S18: Images of half dipstick LFIA strips utilizing Affi-BB-Cys and mAb-3 capture probes alongside AuNP Affi-BB-Cys detection probes. LFIA strips were stored under varying conditions for stability studies. +ve represents the signal at 10 nM and -ve represents the noise utilizing 0 nM Spike trimer. Signal and noise LFIA strip images on days 1, 7, 14, 30, and 90 with strips stored under varying conditions, n = 2. LFIA strips were stored under elevated humidity for 30 days only owing to the harsh environment which could interfere with other assay components.

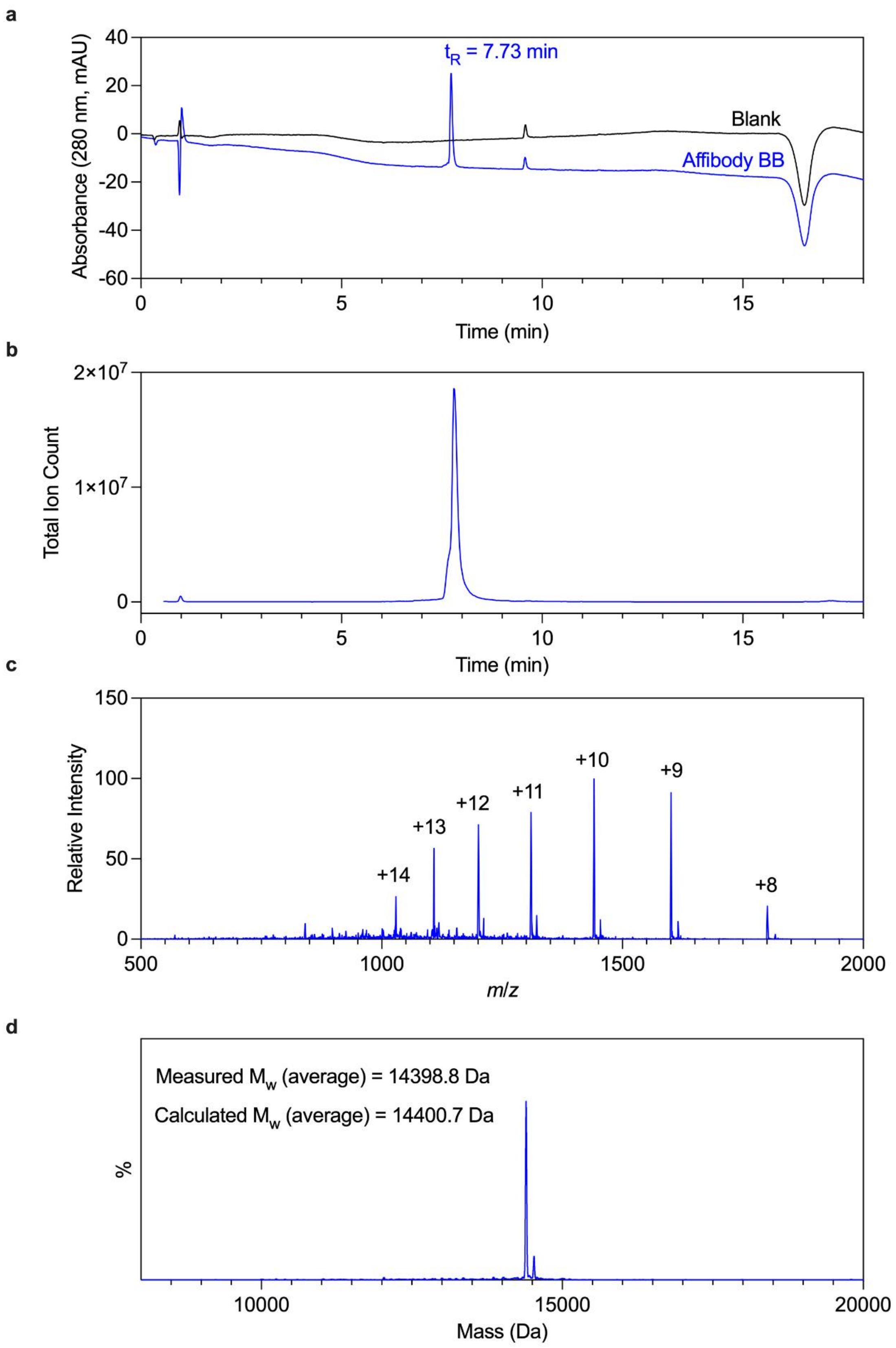

Figure S19: Affibody BB LC-MS analysis and deconvolution. a) HPLC chromatogram of the purified affibody BB (note impurity in blank spectra at ca. 9.2 min). b) Positive mode total ion count. c) Extracted ESI-MS spectra of the peak of interest (tR = 7.73 min). d) Deconvoluted mass using UniDec 6.0.4 demonstrating good agreement with the calculated mass.

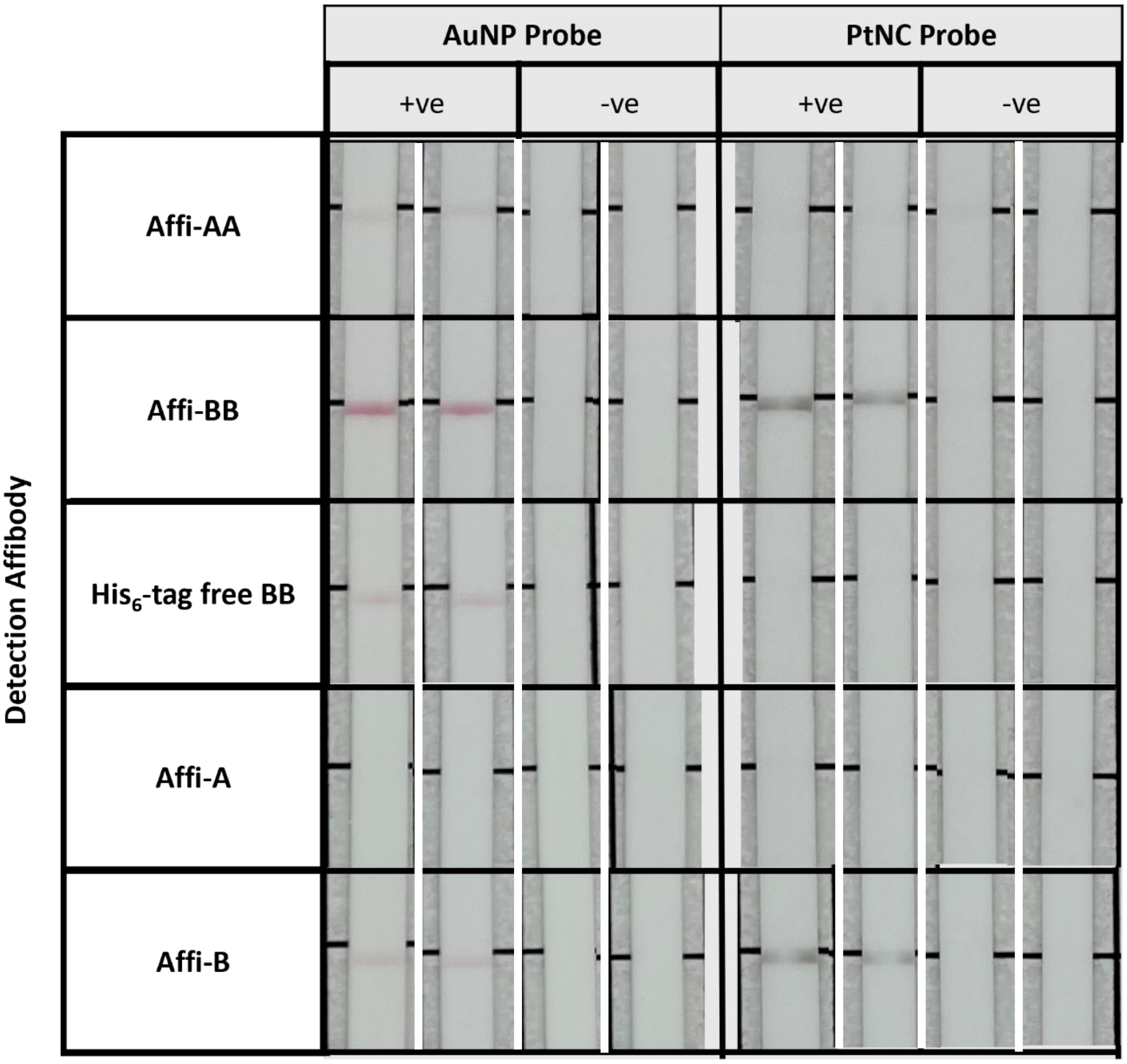

Figure S20: Half dipstick LFIA images utilizing Affi-BB-Cys as a capture probe alongside various affibody detection probes. Figure assembled from various images of LFIA strips. +ve refers to the presence of the Spike trimer antigen, whilst -ve refers to the absence of the target antigen. Left: 40 nm AuNP detection probes showing LFIA strips produced using 2 nM Spike trimer (+ve) or running buffer only (-ve), n = 2. Right: LFIA strips produced using 400 pM Spike trimer (+ve) or PBST only (-ve), with PtNC detection probes, n = 2.

Table S9: Zeta potential measurements of 40 nm AuNP and PtNCs conjugated with His_6-tag free affibody BB. Data given as mean ± standard deviation, n = 3.

Sample	Detection Protein	Zeta Potential/ mV
AuNP Affibody	His_6-tag free Affi-BB	-19.4 ± 3.77
PtNC Affibody	His_6-tag free Affi-BB	-25.9 ± 0.814

Supporting Information Experimental Section

1. *Affibody production and purification*

Tag production of biotinylated His_6-BB-AviTag

Affi-B was subcloned, expressed and purified under native conditions as described in the Materials and Methods section of the main paper, homodimer construct with an N-terminal His_6-tag and C-terminal AviTag. Following purification, the protein was buffer exchanged to PBS with 4.5 mM NH4Ac, and then subjected to enzymatic biotinylation with *E. coli* biotin ligase (BirA). The reaction was performed by incubating 27 µM prepared protein sample with 5 mM ATP, 2.5 mM D-biotin, 5 mM $MgCl_2$ and 3.1 µM BirA enzyme with end-over-end rotation for 1 h at RT and then 2 h at 30 °C. Excess D-biotin was removed by dialysis overnight with at 4 °C with slow stirring against PBS using a dialysis cassette (Slide-A-Lyzer™ Dialysis Cassette, 3.5 MWCO, ThermoFisher Scientific). Biotin labelling of recovered sample was verified by MALDI-MS and SDS-PAGE analyses (binding capacity to streptavidin coated magnetic beads (Dynabeads™ M-280 Streptavidin, Invitrogen) and a streptavidin gel-shift assay).[1]

Production His_6-tag free Affi-BB

A homodimeric construct of Affi-B, with a flexible $(GGGSG)_3$ linker between each affibody monomer unit, was synthesized by Twist Bioscience (Clonal Genes Twist Expression Vector (300 - 500bp), in expression vector pET-28a(+)). The protein was expressed as above. Cells were lysed by sonication and the resulting lysate was heat-treated by boiling for 7 min at 96 °C. The supernatant was buffer exchanged to PBS using a PD-10 desalting column (Cytiva), to remove residual cell debris and medium before purification by Prep-HPLC.

LC-MS analysis

Affibodies were analyzed using an Agilent 1260 Infinity HPLC equipped with a G6130B quadrupole mass spectrometer in ESI mode, G1322A 1260 degasser, G1312B binary pump, G1329B autosampler, G1316A thermostatted column compartment and G7115A DAD WR. HPLC separations were performed using a Phenomenex bioZen™ 2.6 µm WidePore C4, 150 × 2.1 mm LC column (P/No: 00F-4767-AN). Affibodies were diluted to a final concentration of 20 µM in PBS in a HPLC vial containing a low volume insert. The injection volume was 5 µL, flow rate = 0.35 mL min^{-1} and detection wavelengths = 220 nm, 254 nm and 280 nm. The mobile phases were: A = 0.1% formic acid (Sigma) in HPLC grade water (VWR International) and B = 0.1% formic acid in HPLC grade acetonitrile (VWR International). The gradient profile used is described in Table A1. Positive ESI-MS ions were measured from 500 – 2000 m/z. Parent masses were deconvoluted from the positive multiply charged ion mass spectra using UniDec 6.0.4.[2]

Table A1: Gradient profile for LC-MS analyses.

Time/ min	B%
0	2
1.2	2
10.2	98
13.2	98
13.5	2
18	2

Affibody BB purification *via* preparative-HPLC

The His_6-tag free BB affibody was purified preparative-HPLC on a Shimadzu Prominence LC-20A equipped with a Phenomenex Jupiter™ 4 µm Proteo 90 Å, 250 × 10 mm LC column. HPLC purification was performed using a detector wavelength of 220 nm and 280 nm, flow rate = 5 mL min^{-1} and injection loop = 20 mL. The mobile phases were: A = 0.1% formic acid in MilliQ water and B = 0.1% formic acid in HPLC grade acetonitrile. A gradient profile as described in Table A2 was used to purify the affibody of interest. Fractions containing the His_6-tag free BB affibody were confirmed using LC-MS as described above, pooled and lyophilized to give the His_6-tag free BB affibody as a fluffy white powder.

Table A2: Gradient profile for affibody BB purification.

Time/ min	B%
0	5
8	5
32	60
40	60
40.5	5
48	5

2. *Synthesis and Characterization of Nanoparticles and Nanoparticle Conjugates*

Synthesis of platinum nanocatalysts (PtNCs)

Gold nanoparticle seeds with a diameter of ca. 15 nm were synthesized by sodium citrate reduction of $HAuCl_4$ as described by Loynachan *et al*.[3] In a typical synthesis, 10 mL of gold(III) chloride trihydrate aqueous solution (20 mM, Sigma) was added to 180 mL of Ultra Pure Distilled Water (UPDW, Invitrogen) under reflux at 100 °C. The temperature was then reduced to 70 °C, and the reaction initiated by fast injection of 10 mL of trisodium citrate dihydrate (68 mM, Sigma) with vigorous stirring and refluxed for 5 min. The resulting ca. 15 nm gold nanoparticle (AuNP) seeds were cooled to RT and subsequently stored at 4 °C.

120 nm PtNCs were synthesized via the reduction of chloroplatinic acid hydrate on to the 15 nm AuNP seeds.[3] Glassware (24 mL glass vial) was washed 3 x with 10 mL of UPDW before PtNC synthesis. In a typical synthesis, 620 µL of 15 nm AuNP seed (10 nM) was added to a glass vial containing 19.4 mL of UPDW, followed by the addition of 400 µL of 20 w/v% of poly(vinylpyrrolidone) (PVP, MW 10 kDa, Sigma). The solution was briefly vortexed and incubated without stirring for 5 mins. To the polymer coated AuNP seed solution, 800 µL of L-Ascorbic acid (100 mg mL^{-1}, Sigma) was added, followed by the addition of 800 µL of chloroplatinic acid hydrate (100 mM, Sigma). The resulting solution was briefly vortexed and immediately incubated at 65 °C for 45 mins until the color of the solution changed from red to black. PtNCs were then cooled to RT, and excess reagents removed through three sequential washing cycles at 7000 rcf for 5 min with resuspension into UPDW. After the final wash step, PtNCs were resuspended in UPDW and stored at 4 °C.

Dynamic light scattering and zeta potential

Dynamic light scattering and zeta potential measurements were performed on a Zetasizer Nano ZS (Malvern Instruments, Ltd.) equipped with a 633 nm He-Ne laser. Measurement parameters were set using the Zetasizer Nano software v8.02, and samples equilibrated to RT for 120 s before measurements.

Transmission electron microscopy (TEM)

Sample preparation for TEM characterization was performed by diluting PtNCs and AuNPs to a final concentration of 100 pM. The samples were drop-cast onto an ultra-thin carbon support film on lacey carbon grids (Agar Scientific) and left to air dry for 30 mins. TEM imaging was performed using a JEOL 2100F operating at 200 kV, equipped with a Gatan Orius SC1000 camera. TEM micrographs were analyzed using Pebbles (v 2.0) manual fitting method to generate size distribution statistics.[4]

3. *Statistical Analysis*

Determination of Limit of Detection for Nanozyme-LISA Assays

The limit of detection analysis for nanozyme-LISA serial dilution experiments was performed by using the *Detection Limit Fitting Tool* (https://github.com/bensmiller/detection-limit-fitting).[5–7]Briefly, absorbance values were measured at 450 nm (SpectraMax M5 microplate reader (Molecular Devices)), before exporting and assigning the associated S protein concentration. The concentration and absorbance values were imported into the Detection Limit Fitting Tool and a 4-parameter logistic regression fit using the following equation:

$$y = \frac{a - d}{1 + \left(\frac{x}{c}\right)^b} + d$$

where y is the absorption signal, x is the S protein concentration, a is the asymptotes at the blank (background) signal, d is the saturation peak size, b the exponent defining the gradation of the region between the asymptotes (a and d), and c is the dissociation constant equivalent.[6]

The confidence levels (LOD false negative rate, blank false positive rate, variance outlier confidence level, and confidence level for LOD interval) were all set to 5 %.

Determination of Limit of Detection for PtNC LFIA Assays

The limit of detection analysis for nanozyme-LISA serial dilution experiments was performed by using the *Detection Limit Fitting Tool* (https://github.com/bensmiller/detection-limit-fitting).[5–7]Briefly, test line intensities were extracted as previously described, after normalization test line intensities were assigning the associated S protein concentration. The concentration and test line intensity values were imported into the Detection Limit Fitting Tool and a 4-parameter logistic regression fit using the following equation, as defined previously.

The confidence levels (LOD false negative rate, blank false positive rate, variance outlier confidence level, and confidence level for LOD interval) were all set to 5 %.

Determination of Limit of Detection for AuNP LFIA Assays

The limit of detection analysis for nanozyme-LISA serial dilution experiments was performed by using the *Detection Limit Fitting Tool* (https://github.com/bensmiller/detection-limit-fitting).[5–7]Briefly, test line intensities were extracted as previously described, after normalization test lline intensities were assigned to the associated S protein concentration. The concentration and test line intensity values were imported into the Detection Limit Fitting Tool and a Langmuir adsorption model. The Langmuir adsorption model was chosen to fit this data owing to the lower root mean squared error (RMSE), a value that represents the goodness of the fit.[6] The Langmuir adsorption model equation used was as follows:

$$y = \frac{a \cdot 10^x}{c + 10^x} + d$$

where y is the normalized test line signal, x the S protein concentration, a is the saturation peak size, c is the dissociation constant, and d is the blank (background) signal. A $\log_{10}$ transformation of the S protein concentration was used, so the x concentration term was replaced by 10^x.[6]

The confidence levels (LOD false negative rate, blank false positive rate, variance outlier confidence level, and confidence level for LOD interval) were all set to 5 %.

Comparison of Limit of Detection Values: T-test

To compare the statistical significance between two calculated limit of detection values, a T-test was used. The analysis was performed in the *Detection Limit Fitting Tool* (https://github.com/bensmiller/detection-limit-fitting) software.[5] In each case, the limit of detection for the mAb only pair was used a comparison dataset, since this was the previous gold standard assay. The confidence level for the limit of detection interval was set at 5%.

Supporting Information Computational Details

1. *Model construction*

Homology modelling of affibody structures

The experimental amino acid sequences of each affibody (Affi-AA, Affi-BB) were inputted into Basic Local Alignment Search Tool (BLAST)[8] to identify a 3D structural template of the affibodies that could be subsequently used for docking and MD. Staphylococcal protein A (PDB ID: 4NPF)[9] was found as the closest homolog to the target sequences, with its two tandem B domains sharing a high sequence identity overlap with Affi-AA (77.59%) and Affi-BB (79.31%). The MODELLER 10.4 program[10] was used to generate the homology models of each affibody, including the linker that binds the two monomers into a single dimeric affibody. Statistically favorable structures were selected for docking and simulations, as indicated by both the lowest z-DOPE score and the smallest number of Ramachandran outliers as implemented in PROCHECK.[11]

Construction of protein (affibody)–surface models

Protein–surface docking was performed using the DockSurf suite[12] to predict initial configurations of how the affibodies bind to the surface of Au(111), which is the most prevalent and stable crystallographic facet of gold nanoparticles (AuNP). Docking was conducted using the His_6-tag of the affibodies as the anchor point to gold to emulate an experimental scenario of the His_6-tag directing the binding of the protein to the AuNP surface. Favorable affibody–Au orientations were identified using the ΔG_{QM} maps generated in DockSurf, since this strategy of mapping affibody–Au interactions considers both solvation and polarizations effect according to a quantum mechanical level of theory.[12] Then, the original Au(111) surface model from the DockSurf suite was replaced with Au coordinates that are compatible with the Au(111) surface model used in the GolP-CHARMM force field (i.e., slightly altered lattice spacing and containing virtual atom sites).[13] Moreover, to reduce potential bias from the starting orientation, two additional initial orientations of the dimeric affibodies were generated by rotating the monomer with the His_6-tag on the surface of Au(111) at consecutive angles of ~45° relative to the docked structure on the Au(111) surface via the Visual Molecular Dynamics (VMD) program.[14] This resulted in three orientations of the dimeric affibodies on the Au(111) surface: the original structure from DockSurf, a tilted (~45°) structure, and a perpendicular (~90°) structure, relative to the Au(111) surface. All three poses for the homodimeric affibodies were generated with the His_6-tag directly anchored on the Au(111) surface.

Protein–protein (affibody–antigen) docking

The semirigid High Ambiguity Driven protein−protein Docking (HADDOCK) program[15] was employed to create affibody-antigen complexes, composed of A-His_6 and B-His_6 with the receptor binding domain (RBD) of severe acute respiratory syndrome coronavirus 2 (SARS-CoV-2) spike (S) protein (PDB ID: 7R8L).[16] Before docking, all components within the target receptor, except for the RBD were removed. The missing H519 residue was manually added using the BIOVIA Discovery Studio (San Diego, US).[17] Active residues were defined as the two α-helices, containing the mutated affinity modulating residues of A-His_6 and B-His_6, respectively, and the epitope within the RBD of SARS-CoV-2 S protein.[18] The detailed systematic procedure found in reference [15] was then followed with initial randomization of the starting orientation as well as energy minimization, three stages of semi-flexible simulated annealing, and final refinement in 8 Å shell of TIP3P water models to further optimize the conformation within the affibody–antigen interaction site.

2. *Molecular dynamics simulation set up and parameters*

Affibody–surface simulations

Classical MD simulations were carried out using the GROMACS 2018.3 suite[19,20] to model the protein–surface complexes, comprising of the Au(111) slab, dimeric affibodies, citrate anions, explicit aqueous solvent, and salt ions. Intermolecular interactions between the proteins and the citrate-coated Au(111) surface were computed using the GolP-CHARMM force field,[13] which includes dynamic polarization of Au atoms and the CHARMM-modified TIP3P water model.[21] The citrate anions were modelled using the CHARMM-compatible force field developed by Wright, Rodger and Walsh.[22] Consistent with the GolP-CHARMM forcefield, the geometry of the Au atoms was frozen, whereas the Au dipoles were free to rotate on the surface of the gold atoms. All surface systems contained a five-layer gold slab of Au(111), with a unit cell of lateral *xy* dimensions of approximately 120 Å × 120 Å. Periodic boundary conditions were implemented with a vacuum spacer of approximately 200 Å along the perpendicular, *z*-direction from the Au(111) surface to omit any interlayer interactions. The PACKMOL program[23] was utilized to generate the citrate anion surface adlayers at a concentration of ~3×10^{-10} mol/cm^2. This concentration of the citrate anions was chosen based upon a previous study, demonstrating an approximate coverage of 45%.[24] Systems were solvated with a water density of ~1 g/cm^3, a NaCl salt concentration of 0.15 M, as well as additional NaCl counterions to ensure charge neutrality. In all simulations, bond lengths were constrained using the LINCS algorithm[25], with long-range electrostatic forces calculated using the particle-mesh Ewald scheme (PME)[26] and a grid spacing of 0.12 nm, whereas cutoff radius of 1.2 nm for Coulomb and van der Waals potentials were selected for the calculation of short-range nonbonded interactions.

Energy minimization was implemented using the steepest-descent gradient method for a maximum of 100,000 steps. This was followed by 20 ns of molecular dynamics in the canonical NVT ensemble at 298 K, employing flat-bottomed potentials to restrain desorption of citrate from gold while gently equilibrating the solvent and the citrate adlayer on Au(111) (Table A3). During this equilibration process, the heavy atoms of the affibody were also restrained along all three cartesian coordinates with harmonic position restraints (Table A3).

Table A3. Citrate adlayer equilibration protocol for affibody–citrate–Au(111) systems.

Simulation time/ ns	Force constant*/ kJ mol^{-1} nm^{-2}	Threshold citrate–Au(111) distance relative to the center of mass/ nm	Thermostat
5	1000	0.5	Berendsen[27]
5	500*	0.5	Berendsen[27]
5	500*	1.0	Nose-Hoover[28,29]
5	250*	1.0	Nose-Hoover[28,29]

*Harmonic restraints with equivalent force constant applied to protein heavy atoms

Final production runs were conducted in triplicate for 500 ns at 298 K using the Nose-Hoover thermostat [28,29] and an integration timestep of 1 fs. In these simulations, no restraints were applied to citrate, but a flat-bottom potential was maintained between the last histidine residue and the gold surface, with a force constant of 1,000 kJ mol^{-1} nm^{-2} and a threshold value of 0.5 nm. This was carried out to mimic the laboratory setup having His$_6$-tag bound affibodies on the surface of AuNP.

Simulations of affibodies in solution and affibody–antigen complexes

MD simulations were performed using the above GROMACS program, in conjunction with the CHARMM27 forcefield.[19,20,30] TIP3P water molecules were used to solvate each system in a dodecahedral box with a minimum distance of 2.0 nm between the edge of the box and any protein atoms. Salt (0.15 M) and counter ions were added to neutralize the system. Energy minimization for

100,000 steps via the steepest-descent gradient protocol was then implemented for each system. Throughout all simulations, bond lengths were constrained using the LINCS algorithm[25], enabling an integration timestep of 2 fs. Preliminary runs, consisting of NVT and isothermal–isobaric (NPT) ensembles were conducted where each structure was restrained for 500 ps using the Berendsen thermostat and barostat.[27] Long-range electrostatic forces were calculated *via* the particle-mesh Ewald scheme (PME)[26] (grid spacing 0.12 nm). Short-range nonbonded interactions were cutoff at a radius of 1.2 nm. In triplicate, 500 ns of unrestrained simulations were run for affibodies in solution, whereas 200 ns of final MD simulations were executed for affibody–antigen complexes. During these simulations, temperature and pressure are maintained at 298 K and 1.0 bar, respectively using the V-rescale[31] thermostat and Parrinello-Rahman barostat.[32–34] Initial atomic velocities were randomly assigned according to a Maxwell distribution.

3. *Supplementary Computational Results*

The following is a discussion of affibody dimers in the form [affibody]-linker-[affibody]-His_6 constructs, ordered from N to C terminus, as produced.

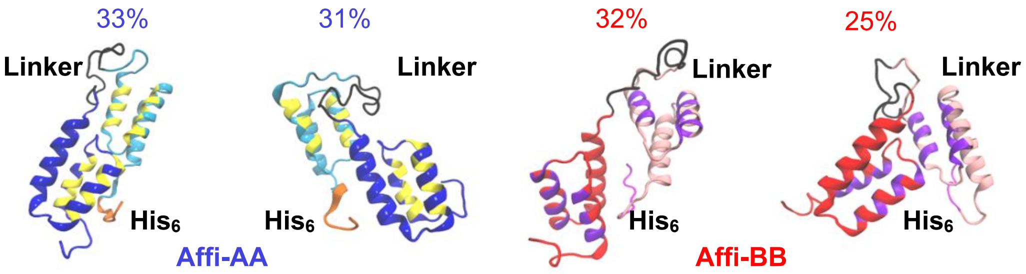

Figure S21: Representative median structures of heteromeric affibodies obtained from the top two populated cluster analysis for dimeric affibody constructs in aqueous solution. Blue and red colors refer to the N-terminal affibody in Affi-AA and Affi-BB, respectively, while cyan and pink colors refer to the C-terminal affibody in Affi-AA and Affi-BB, respectively. Mutated affinity modulating residues between dimeric affibody constructs are colored in yellow for Affi-AA and purple for Affi-BB.

Figure S21 shows the representative median structures of Affi-AA and Affi-BB in aqueous solutions. Representative median structures of dimeric affibodies showed that the mutated affinity modulating residues within the N-terminal affibody of both Affi-AA and Affi-BB are exposed for an antigen to interact. For the C-terminal affibody, the presence of the linker moiety may limit binding to an antigen.

Table S10: Contact probabilities of mutated affinity modulating residues between dimeric affibody constructs with the linker or His_6-tag in aqueous solution.

Affibody Dimer	N-terminal affibody (His_6-tag free)	Contact probability	C-terminal affibody (His_6 - tagged)	Contact probability
		Linker		Linker
Affi-AA	A	0.00	A-His_6	0.98
Affi-BB	B	0.00	B-His_6	0.67

Table S10 presents an analysis of the contacts formed between the linker and both the C-terminal and the N-terminal affibody in aqueous solution. The results indicate that the mutated affinity modulating residues in the N-terminal monomer of Affi-AA and Affi-BB do not form contacts with the linker. This suggests the N-terminal affibody's affinity modulating residues are more available for interaction with an antigen compared to the equivalent residues on the C-terminal His_6-tagged monomer. This is supported by the median structures shown in Figure S22, illustrating that the C-terminal affibody of both Affi-AA and Affi-BB is available for antigen binding.

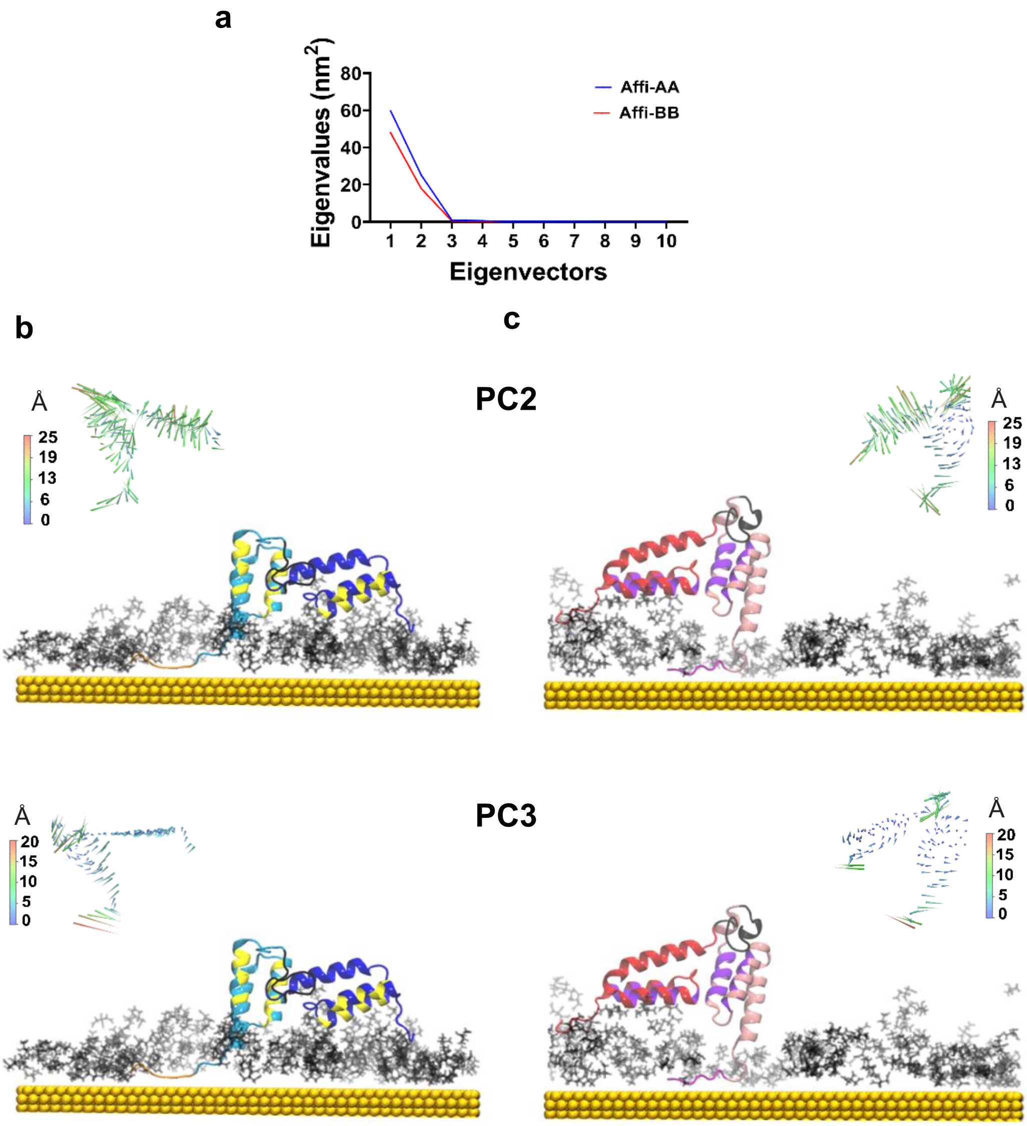

Figure S22: a) Top 10 eigenvalues for Affi-AA and Affi-BB. b, c) Porcupine plots illustrating the magnitude of dynamic motion in Affi-AA (b) and Affi-BB (c). Blue/red refers to N-terminal affibody (without the His_6-tag), while cyan/pink refers to C-terminal affibody (with the His_6-tag). Mutated affinity modulating residues between dimeric affibody constructs are colored in yellow for Affi-AA and purple for Affi-BB. The inset porcupine plots illustrate the magnitude of the protein residue dynamics. Au surface atoms shown in dark yellow, citrate molecules in grey. Water and ions not shown for clarity. PC2 represents 28% of the movement for Affi-AA and 26% of the movement for Affi-BB, with respective cosine value of 0.52 and 0.44. PC3 represents 1% of the movement for Affi-AA and Affi-BB, with respective cosine values of 0.01 and 0.06.

Principal component analysis (PCA) was used to examine the motion of Affi-AA and Affi-BB by filtering out residue movements that are associated with Brownian and/or random motion in the MD trajectories. Figure S22a shows contributions of the first 10 eigenvalues to the observed movements in both constructs. The first principal component (PC) has the largest eigenvalue—approximately 60 nm^2 for Affi-AA and 50 nm^2 for Affi-BB—indicating that affibody motion is predominantly captured by the first PC. The second and third PCs contribute to lateral movement for Affi-AA and Affi-BB on the

surface of citrate-coated Au(111), with the second PC showing eigenvalues of ~40nm^2 for Affi-AA and ~30nm^2 for Affi-BB, and the third PC contributing ~0.2 nm^2 for both constructs. Figures S22b and S22c display porcupine plots that depict the motion of Affi-AA and Affi-BB along the second and third eigenvectors. Results from the second and third PCs generally align with the first PC (Main Text Figure 5h and 5i), with porcupine plots of the protein backbone alpha-carbon atoms (Cα), depicting the movement of the residues along the second and third eigenvectors (PC2 & PC3). The C-terminal affibody is perpendicular to the Au(111) surface, allowing for antigen binding, while the N-terminal monomer, away from the His$_6$-tag, is parallel to surface, with mutated affinity modulating residues orientated towards Au(111). Low cosine values for each PC suggest that the projected motion is not likely arbitrary, indicating essential motions of both dimeric affibody constructs on the citrate-coated Au(111) surface.[35]

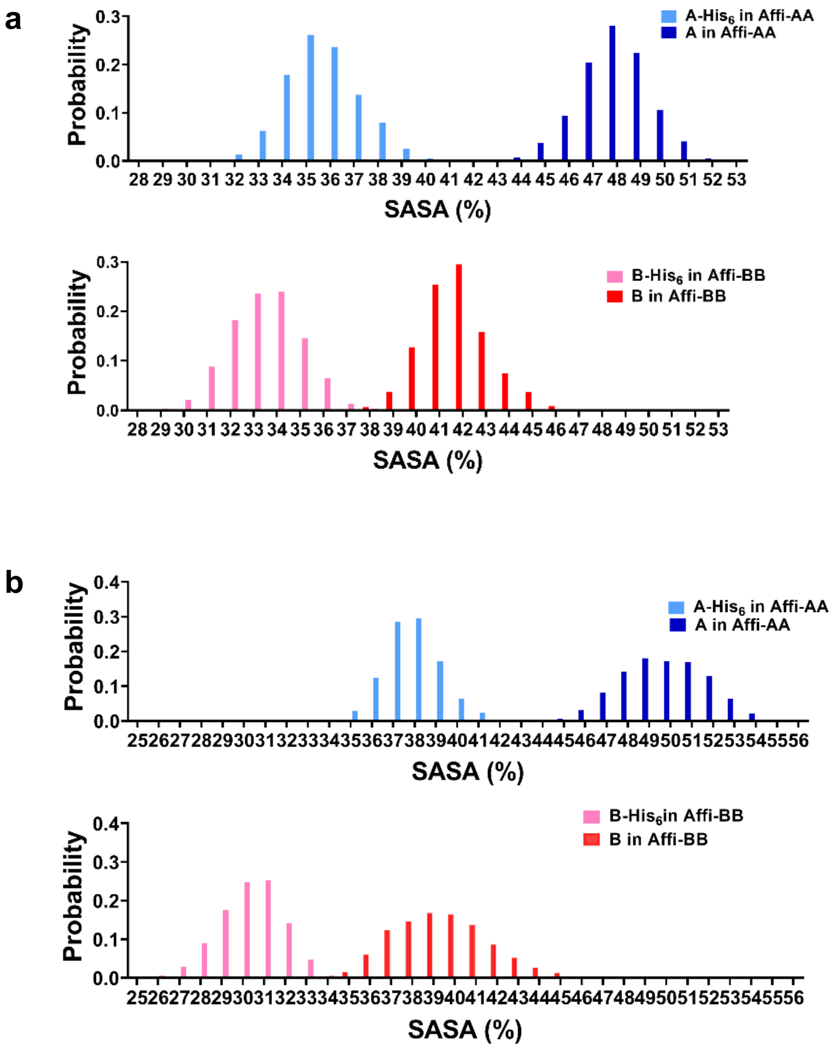

Figure S23: Distribution of normalized solvent-accessible surface area (SASA) for the mutated affinity modulating residues between dimeric affibody constructs in aqueous solutions (a) and on the surface of citrate-coated Au(111) (b). Normalization was performed by dividing the SASA values by the maximum exposure observed in the respective system. Blue/red colors refer to the N-terminal affibody, while cyan/pink colors refer to the C-terminal affibody.

Figure S23 shows the SASA of mutated affinity modulating residues on the surface of Au(111) and in aqueous solution. The C-terminal affibodies all presented a lower SASA compared to the N-terminal affibody, which could be due to the higher probability of contact formation with the linker for the C-terminal affibody (Table S10, refer also to Table S12). Nonetheless, the C-terminal affibody of Affi-BB offers a more favorable binding to an antigen than that of Affi-AA due to its more open surface bound conformation facilitated by the repulsive interactions and the flexibility of the linker as discussed in the main text of the manuscript.

Table S11: Comparative average distance analysis of mutated affinity modulating residues between dimeric affibody constructs and the third helix to the surface of Au(111).

Affibody dimer	N-terminal affibody (His_6-tag free)		C-terminal affibody (His_6-tagged)	
	Distance of affinity residues to Au(111) (Å)	Distance of 3rd Helix to Au(111) (Å)	Distance of affinity residues to Au(111) (Å)	Distance of 3rd Helix to Au(111) (Å)
Affi-AA	13 ± 2	19 ± 4	25 ± 3	10 ± 4
Affi-BB	16 ± 2	24 ± 3	21 ± 2	10 ± 4

**Values are expressed as mean ± standard error of the mean for the minimum separation distance between the Au(111) surface and the center of mass for affinity modulated residues in an affibody monomer.*

Table S12: Contact probabilities of mutated affinity modulating residues between dimeric affibody constructs with the linker or citrate anions on the surface of Au(111).

Affibody dimer	N-terminal affibody (His_6-tag free) contact probability		C-terminal affibody (His_6-tagged) contact probability		His_6 contact probability
	Linker	Citrate anions	Linker	Citrate anions	Citrate anions
Affi-AA	0.01	0.86	1.00	0.04	0.85
Affi-BB	0.33	0.90	0.99	0.27	0.84

Table S11 and Table S12 establish quantitative metrics describing steric availability of the mutated affinity modulating residues. Table S11 compares the distance from mutated affinity modulating residues of each monomer and the third helix (not comprising any mutated affinity modulating residues) to the surface of Au(111). In both Affi-AA and Affi-BB systems, the C-terminal affibody has its mutated affinity modulating residues further away from the surface, whereas the N-terminal affibody shows the opposite trend. Table S12 shows the contact probabilities between the affibody units and the $(GGGSG)_3$ linker and citrate anions. Contact analysis with citrate anions supports the affibody–surface distance analysis, demonstrating that the N-terminal affibody monomers form contacts with citrate anions (probability >0.80), whereas for the C-terminal affibody contacts are much less likely (< 0.30). This is due to the differing orientation between the N-terminal affibody monomer and the C-terminal affibody monomer, where the N-terminal affibody monomer as indicated by the PCA orientates downwards toward Au(111) surface. Additionally, for both Affi-AA and Affi-BB, residues in the C-terminal affibody were found to form stable contacts with the linker (probability >0.99). Nevertheless, as the C-terminal affibody has its mutated affinity modulating residues further away

from the surface, it is more likely that it is more accessible for antigen to bind compared to the N-terminal affibody, supporting the results of the PCA and porcupine plots.

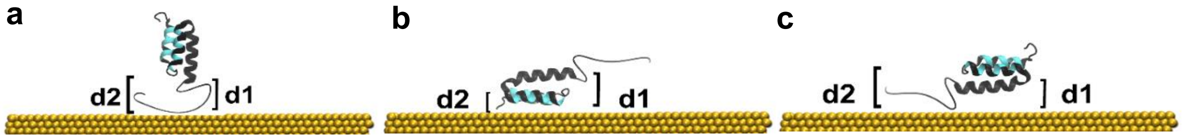

Figure S24: Exemplary cartoon depictions of three possible orientations of the monomeric affibody model with His_6 on the surface of citrate-coated Au(111). a) shows the perpendicular orientation, b) illustrates the parallel orientation with the affinity modulating residues facing towards Au(111), and c) depicts the parallel orientation with affinity modulating residues facing away from Au(111). Mutated affinity modulating residues are colored blue. Distance d1 measures the minimum distance of the third helix to the Au(111) surface. Distance d2 measures the minimum distance from the first and second helices to the Au(111) surface. For clarity, citrate anions and solvent are not shown.

Distance measurements of specific helices from the Au(111) surface are used to determine the adsorption orientation of the affibody. The analysis compares the distance of the third helix (d1), which lacks mutated affinity modulating residues, with the distance of the mutated affinity residues, within the first and second helices (d2), relative to the Au(111) surface. Figure S24 illustrates three possible affibody adsorption orientations. In perpendicular orientations (Figure S24a), the magnitude of d2 is slightly larger than d1, with the mutated affinity modulating residues solvent exposed and accessible for antigen binding. In parallel orientations, if the first and second helices are oriented towards the surface (Figure S24b), i.e. d1 > d2, the mutated affinity modulating residues will be sterically unavailable for antigen binding due to their proximity to the surface. However, if the third helix sits lower than the first two helices (d1 < d2), mutated affinity modulating residues will be accessible for antigen binding (Figure S24c).

Table S13: Average binding energy of monomeric affibodies to the RBD of SARS-CoV-2 S protein.

**Energy**	**Binding of the monomeric A-His$_6$ affibody to the RBD*/ kJ mol^{-1}**	**Binding of the monomeric B-His$_6$ affibody to the RBD*/ kJ mol^{-1}**
Van der Waals	-313.60 ± 0.2	-272.80 ± 0.2
Electrostatic	-21.73 ± 0.05	-28.50 ± 0.05
Polar Solvation	278.40 ± 0.5	205.6 ± 0.5
Non-Polar Solvation	-42.01 ± 0.03	-32.72 ± 0.03
**Total Binding Energy**	**-98.90 ± 0.5**	**-128.50 ± 0.5**

*Energies are shown as mean ± standard error of the mean

Table S13 compares the average binding energy of the A-His$_6$ affibody and B-His$_6$ affibody monomers to the RBD of SARS-CoV-2 S protein. In general, both monomers could bind to the antigen, as shown by the negative value. Nonetheless, the B-His$_6$ affibody monomer binds stronger than the A-His$_6$ affibody monomer (-128.50 ± 0.5 kJ mol^{-1} compared to -98.90 ± 0.5 kJ mol^{-1}). Van der Waals and electrostatic interactions, as well as non-polar solvation contribute to favorable binding, while polar solvation hinders the formation of protein–protein complexes. Overall, the findings indicated that both monomers could interact favorably with the RBD of SARS-CoV-2 S protein, and that the B-His$_6$ affibody is potentially a stronger binder than the A-His$_6$ affibody.

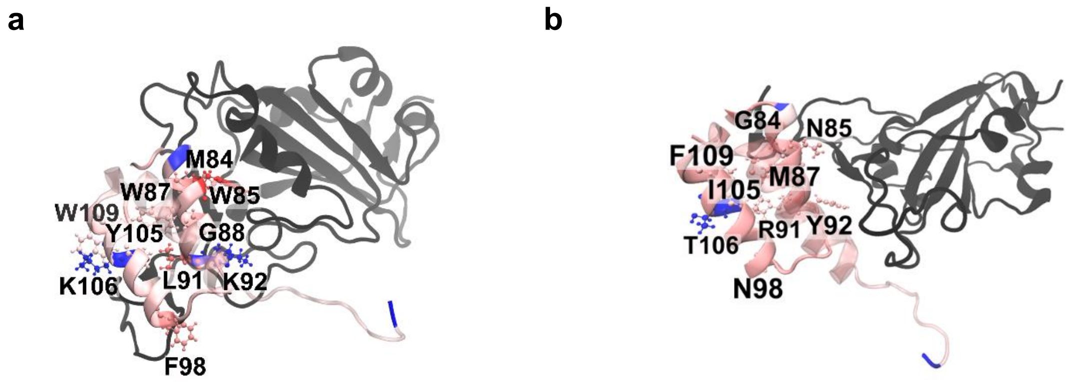

Figure S25: Cartoon representations of the binding of A-His$_6$ **(a)** and B-His$_6$ **(b)** to the RBD of SARS-CoV-2 S protein. Residues are colored according to their binding energies in kJ mol^{-1}. Red denotes a more favorable binding, whilst blue denotes a more unfavorable binding.

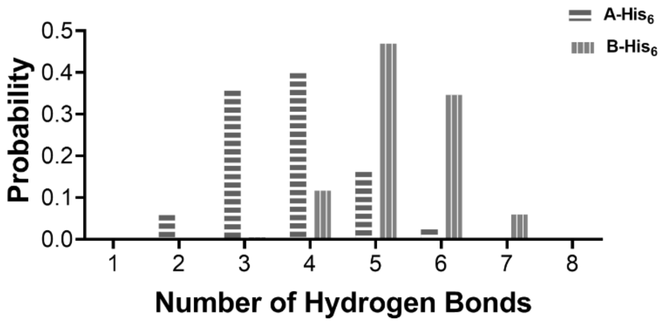

Figure S26: Comparative number of hydrogen bond analysis of the A-His$_6$ (horizonal lines) and B-His$_6$ (vertical lines) to the RBD of SARS-CoV-2 S protein.

Figure S26 compares the number of occurring hydrogen bonds between the A-His$_6$ and B-His$_6$ and the RBD. The median number of hydrogen bonds forming between A-His$_6$ and the RBD is lower than that observed for B-His$_6$ and the RBD. This is consistent with the MM-PBSA calculation, indicating that B-His$_6$ exhibits stronger binding to the RBD than the A-His$_6$.

References:

(1) Fairhead, M.; Howarth, M. Site-Specific Biotinylation of Purified Proteins Using BirA; 2015; pp 171–184. https://doi.org/10.1007/978-1-4939-2272-7_12.

(2) Marty, M. T.; Baldwin, A. J.; Marklund, E. G.; Hochberg, G. K. A.; Benesch, J. L. P.; Robinson, C. V. Bayesian Deconvolution of Mass and Ion Mobility Spectra: From Binary Interactions to Polydisperse Ensembles. *Anal Chem* **2015**, *87* (8), 4370–4376. https://doi.org/10.1021/acs.analchem.5b00140.

(3) Loynachan, C. N.; Thomas, M. R.; Gray, E. R.; Richards, D. A.; Kim, J.; Miller, B. S.; Brookes, J. C.; Agarwal, S.; Chudasama, V.; McKendry, R. A.; Stevens, M. M. Platinum Nanocatalyst Amplification: Redefining the Gold Standard for Lateral Flow Immunoassays with Ultrabroad Dynamic Range. *ACS Nano* **2018**, *12* (1). https://doi.org/10.1021/acsnano.7b06229.

(4) Mondini, S.; Ferretti, A. M.; Puglisi, A.; Ponti, A. Pebbles and PebbleJuggler: Software for Accurate, Unbiased, and Fast Measurement and Analysis of Nanoparticle Morphology from Transmission Electron Microscopy (TEM) Micrographs. *Nanoscale* **2012**, *4* (17), 5356. https://doi.org/10.1039/c2nr31276j.

(5) Ben Miller. *Detection Limit Fitting Tool (https://github.com/bensmiller/detection-limit-fitting), GitHub.* 2022. https://github.com/bensmiller/detection-limit-fitting (accessed 2024-05-28).

(6) Miller, B. S.; Thomas, M. R.; Banner, M.; Kim, J.; Chen, Y.; Wei, Q.; Tseng, D. K.; Göröcs, Z. S.; Ozcan, A.; Stevens, M. M.; McKendry, R. A. Sub-Picomolar Lateral Flow Antigen Detection with Two-Wavelength Imaging of Composite Nanoparticles. *Biosens Bioelectron* **2022**, *207*, 114133. https://doi.org/10.1016/j.bios.2022.114133.

(7) Holstein, C. A.; Griffin, M.; Hong, J.; Sampson, P. D. Statistical Method for Determining and Comparing Limits of Detection of Bioassays. *Anal Chem* **2015**, *87* (19), 9795–9801. https://doi.org/10.1021/acs.analchem.5b02082.

(8) Altschul, S. F.; Gish, W.; Miller, W.; Myers, E. W.; Lipman, D. J. Basic Local Alignment Search Tool. *J Mol Biol* **1990**, *215* (3), 403–410. https://doi.org/10.1016/S0022-2836(05)80360-2.

(9) Deis, L. N.; Pemble, C. W.; Qi, Y.; Hagarman, A.; Richardson, D. C.; Richardson, J. S.; Oas, T. G. Multiscale Conformational Heterogeneity in Staphylococcal Protein A: Possible Determinant of Functional Plasticity. *Structure* **2014**, *22* (10), 1467–1477. https://doi.org/10.1016/j.str.2014.08.014.

(10) Šali, A.; Blundell, T. L. Comparative Protein Modelling by Satisfaction of Spatial Restraints. *J Mol Biol* **1993**, *234* (3), 779–815. https://doi.org/10.1006/jmbi.1993.1626.

(11) Laskowski, R. A.; MacArthur, M. W.; Moss, D. S.; Thornton, J. M. PROCHECK: A Program to Check the Stereochemical Quality of Protein Structures. *J Appl Crystallogr* **1993**, *26* (2), 283–291. https://doi.org/10.1107/S0021889892009944.

(12) Barbault, F.; Brémond, E.; Rey, J.; Tufféry, P.; Maurel, F. DockSurf: A Molecular Modeling Software for the Prediction of Protein/Surface Adhesion. *J Chem Inf Model* **2023**, *63* (16), 5220–5231. https://doi.org/10.1021/acs.jcim.3c00569.

(13) Wright, L. B.; Rodger, P. M.; Corni, S.; Walsh, T. R. GolP-CHARMM: First-Principles Based Force Fields for the Interaction of Proteins with Au(111) and Au(100). *J Chem Theory Comput* **2013**, *9* (3), 1616–1630. https://doi.org/10.1021/ct301018m.

(14) Humphrey, W.; Dalke, A.; Schulten, K. VMD: Visual Molecular Dynamics. *J Mol Graph* **1996**, *14* (1), 33–38. https://doi.org/10.1016/0263-7855(96)00018-5.

(15) Dominguez, C.; Boelens, R.; Bonvin, A. M. J. J. HADDOCK: A Protein−Protein Docking Approach Based on Biochemical or Biophysical Information. *J Am Chem Soc* **2003**, *125* (7), 1731–1737. https://doi.org/10.1021/ja026939x.

(16) Muecksch, F.; Weisblum, Y.; Barnes, C. O.; Schmidt, F.; Schaefer-Babajew, D.; Wang, Z.; C. Lorenzi, J. C.; Flyak, A. I.; DeLaitsch, A. T.; Huey-Tubman, K. E.; Hou, S.; Schiffer, C. A.; Gaebler, C.; Da Silva, J.; Poston, D.; Finkin, S.; Cho, A.; Cipolla, M.; Oliveira, T. Y.; Millard, K. G.; Ramos, V.; Gazumyan, A.; Rutkowska, M.; Caskey, M.; Nussenzweig, M. C.; Bjorkman, P. J.; Hatziioannou, T.; Bieniasz, P. D. Affinity Maturation of SARS-CoV-2 Neutralizing Antibodies Confers Potency, Breadth, and Resilience to Viral Escape Mutations. *Immunity* **2021**, *54* (8), 1853-1868.e7. https://doi.org/10.1016/j.immuni.2021.07.008.

(17) BIOVIA, D. S. Discovery Studio Modeling Environment. San Diego, 2021.

(18) Lan, J.; Ge, J.; Yu, J.; Shan, S.; Zhou, H.; Fan, S.; Zhang, Q.; Shi, X.; Wang, Q.; Zhang, L.; Wang, X. Structure of the SARS-CoV-2 Spike Receptor-Binding Domain Bound to the ACE2 Receptor. *Nature* **2020**, *581* (7807), 215–220. https://doi.org/10.1038/s41586-020-2180-5.

(19) Abraham, M. J.; Murtola, T.; Schulz, R.; Páll, S.; Smith, J. C.; Hess, B.; Lindahl, E. GROMACS: High Performance Molecular Simulations through Multi-Level Parallelism from Laptops to Supercomputers. *SoftwareX* **2015**, *1–2*, 19–25. https://doi.org/10.1016/j.softx.2015.06.001.

(20) Hess, B.; Kutzner, C.; van der Spoel, D.; Lindahl, E. GROMACS 4: Algorithms for Highly Efficient, Load-Balanced, and Scalable Molecular Simulation. *J Chem Theory Comput* **2008**, *4* (3), 435–447. https://doi.org/10.1021/ct700301q.
(21) Jorgensen, W. L.; Chandrasekhar, J.; Madura, J. D.; Impey, R. W.; Klein, M. L. Comparison of Simple Potential Functions for Simulating Liquid Water. *J Chem Phys* **1983**, *79* (2), 926–935. https://doi.org/10.1063/1.445869.
(22) Wright, L. B.; Rodger, P. M.; Walsh, T. R. Aqueous Citrate: A First-Principles and Force-Field Molecular Dynamics Study. *RSC Adv* **2013**, *3* (37), 16399. https://doi.org/10.1039/c3ra42437e.
(23) Martínez, L.; Andrade, R.; Birgin, E. G.; Martínez, J. M. P <scp>ACKMOL</Scp> : A Package for Building Initial Configurations for Molecular Dynamics Simulations. *J Comput Chem* **2009**, *30* (13), 2157–2164. https://doi.org/10.1002/jcc.21224.
(24) Park, J.-W.; Shumaker-Parry, J. S. Structural Study of Citrate Layers on Gold Nanoparticles: Role of Intermolecular Interactions in Stabilizing Nanoparticles. *J Am Chem Soc* **2014**, *136* (5), 1907–1921. https://doi.org/10.1021/ja4097384.
(25) Hess, B.; Bekker, H.; Berendsen, H. J. C.; Fraaije, J. G. E. M. LINCS: A Linear Constraint Solver for Molecular Simulations. *J Comput Chem* **1997**, *18* (12), 1463–1472. https://doi.org/10.1002/(SICI)1096-987X(199709)18:12<1463::AID-JCC4>3.0.CO;2-H.
(26) Essmann, U.; Perera, L.; Berkowitz, M. L.; Darden, T.; Lee, H.; Pedersen, L. G. A Smooth Particle Mesh Ewald Method. *J Chem Phys* **1995**, *103* (19), 8577–8593. https://doi.org/10.1063/1.470117.
(27) Berendsen, H. J. C.; Postma, J. P. M.; van Gunsteren, W. F.; DiNola, A.; Haak, J. R. Molecular Dynamics with Coupling to an External Bath. *J Chem Phys* **1984**, *81* (8), 3684–3690. https://doi.org/10.1063/1.448118.
(28) Hoover, W. G. Canonical Dynamics: Equilibrium Phase-Space Distributions. *Phys Rev A (Coll Park)* **1985**, *31* (3), 1695–1697. https://doi.org/10.1103/PhysRevA.31.1695.
(29) Nosé, S. A Molecular Dynamics Method for Simulations in the Canonical Ensemble. *Mol Phys* **1984**, *52* (2), 255–268. https://doi.org/10.1080/00268978400101201.
(30) Bjelkmar, P.; Larsson, P.; Cuendet, M. A.; Hess, B.; Lindahl, E. Implementation of the CHARMM Force Field in GROMACS: Analysis of Protein Stability Effects from Correction Maps, Virtual Interaction Sites, and Water Models. *J Chem Theory Comput* **2010**, *6* (2), 459–466. https://doi.org/10.1021/ct900549r.
(31) Bussi, G.; Donadio, D.; Parrinello, M. Canonical Sampling through Velocity Rescaling. *J Chem Phys* **2007**, *126* (1). https://doi.org/10.1063/1.2408420.
(32) Parrinello, M.; Rahman, A. Crystal Structure and Pair Potentials: A Molecular-Dynamics Study. *Phys Rev Lett* **1980**, *45* (14), 1196–1199. https://doi.org/10.1103/PhysRevLett.45.1196.
(33) Parrinello, M.; Rahman, A. Polymorphic Transitions in Single Crystals: A New Molecular Dynamics Method. *J Appl Phys* **1981**, *52* (12), 7182–7190. https://doi.org/10.1063/1.328693.
(34) Parrinello, M.; Rahman, A. Strain Fluctuations and Elastic Constants. *J Chem Phys* **1982**, *76* (5), 2662–2666. https://doi.org/10.1063/1.443248.
(35) Hess, B. Convergence of Sampling in Protein Simulations. *Phys Rev E* **2002**, *65* (3), 031910. https://doi.org/10.1103/PhysRevE.65.031910.
